# Kidney tissue regeneration using bioactive scaffolds incorporated with differentiating extracellular vesicles and intermediate mesoderm cells

**DOI:** 10.1186/s40824-023-00471-x

**Published:** 2023-12-05

**Authors:** Seung-Gyu Cha, Won-Kyu Rhim, Jun Yong Kim, Eun Hye Lee, Seung Yeon Lee, Jeong Min Park, Jeoung Eun Lee, Hyeji Yoon, Chun Gwon Park, Bum Soo Kim, Tae Gyun Kwon, Youngmi Lee, Dong Ryul Lee, Dong Keun Han

**Affiliations:** 1 Department of Biomedical Science, CHA University, 335 Pangyo-ro, Bundang-gu, Seongnam- si, 13488 Gyeonggi-do Republic of Korea; 2 Department of Biomedical Engineering, SKKU Institute for Convergence, Sungkyunkwan University (SKKU), 2066 Seobu-ro, Jangan-gu, Suwon-si, 16419 Gyeonggi-do Republic of Korea; 3 Intelligent Precision of Healthcare Convergence, SKKU Institute for Convergence, Sungkyunkwan University (SKKU), 2066 Seobu-ro, Jangan-gu, Suwon-si, 16419 Gyeonggi-do Republic of Korea; 4 Joint Institute for Regenerative Medicine, Kyungpook National University, Jung-gu, Daegu, 41944 Republic of Korea; 5 Bundang Medical Center, CHA Advanced Research Institute, CHA University, Sungnam- si, 13488 Gyeonggi-do Republic of Korea; 6 Department of Urology, School of Medicine, Kyungpook National University, Jung-gu, Daegu, 41944 Republic of Korea; 7 Department of Chemistry and Nanoscience, Ewha Womans University, Seodaemun-gu, Seoul, Republic of Korea

**Keywords:** Kidney tissue regeneration, PMEZ scaffold, Intermediate mesoderm (IM), Differentiating extracellular vesicle (dEV), Kidney differentiation

## Abstract

**Background:**

To overcome the limitations of current alternative therapies for chronic kidney disease (CKD), tissue engineering-mediated regeneration strategies have demonstrated the possibilities for complete kidney tissue regeneration. Given the challenges associated with the reproducibility of renal basal cells, the incorporation of intermediate mesoderm (IM) cells and bioactive materials to control bioactivities of cells with supported scaffolds should be considered as a viable approach to enable the regeneration of the complex kidney structure via renal differentiation.

**Methods:**

We developed PMEZ scaffolds by combining crucial bioactive components, such as ricinoleic acid-grafted Mg(OH)_2_ (M), extracellular matrix (E), and alpha lipoic acid-conjugated ZnO (Z) integrated into biodegradable porous PLGA (P) platform. Additionally, we utilized differentiating extracellular vesicles (dEV) isolated during intermediate mesoderm differentiation into kidney progenitor cells, and IM cells were serially incorporated to facilitate kidney tissue regeneration through their differentiation into kidney progenitor cells in the 3/4 nephrectomy mouse model.

**Results:**

The use of differentiating extracellular vesicles facilitated IM differentiation into kidney progenitor cells without additional differentiation factors. This led to improvements in various regeneration-related bioactivities including tubule and podocyte regeneration, anti-fibrosis, angiogenesis, and anti-inflammation. Finally, implanting PMEZ/dEV/IM scaffolds in mouse injury model resulted in the restoration of kidney function.

**Conclusions:**

Our study has demonstrated that utilizing biodegradable PLGA-based scaffolds, which include multipotent cells capable of differentiating into various kidney progenitor cells along with supporting components, can facilitate kidney tissue regeneration in the mouse model that simulates CKD through 3/4 nephrectomy.

**Graphical Abstract:**

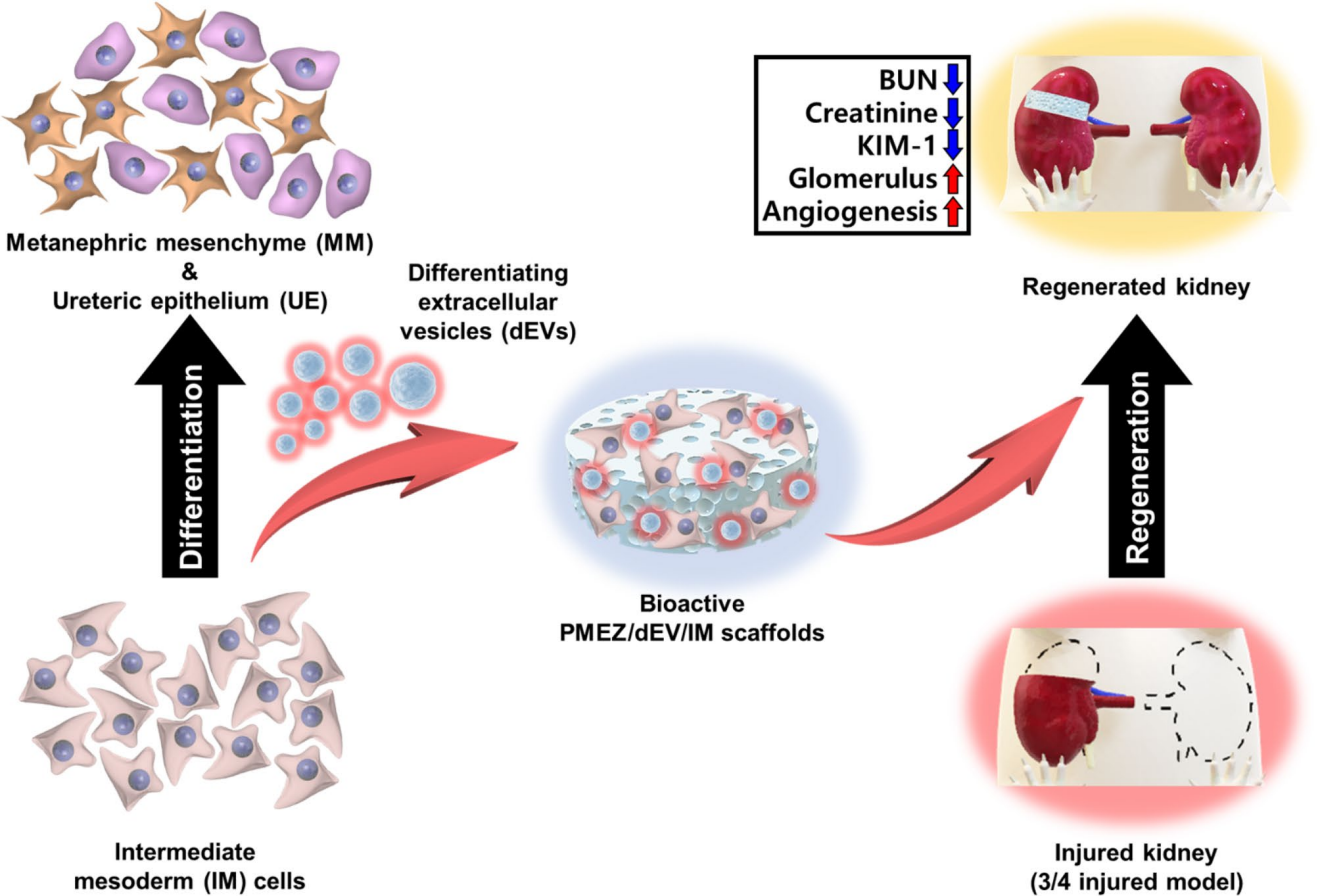

**Supplementary Information:**

The online version contains supplementary material available at 10.1186/s40824-023-00471-x.

## **Background**

Chronic kidney disease (CKD) is a condition in which the kidneys are injured, leading to irreversible structural and functional damage. It has become a global public health problem with increasing incidence rates [1]. Although two types of therapeutic strategies, dialysis and transplantation, have been used to treat patients with end-stage kidney disease (ESKD), both treatments have their limitations [2]. Dialysis is a temporary method that cannot fully replace kidney function and can lead to complications such as cardiovascular disease and anemia. Kidney transplantation also has limitations, including a shortage of donors and the need for continuous use of immunosuppressants after transplantations [3]. With increasing demands for therapeutics of CKD, there has been a focus on tissue engineering and regenerative medicine strategies to reconstruct kidney structures and restore kidney function. While current studies have not yet achieved complete kidney regeneration, cell-based therapeutic approaches have been actively explored based on the cellular mechanisms of regeneration. Specifically, various types of kidney progenitor cells have been investigated as potential regeneration strategies due to the relatively low reproducibility of renal basal cells in adult kidney tissues [4, 5]. The nephron, which is the structural and functional unit of the kidney, consists of numerous cell types and is only generated only during nephrogenesis in the developmental processes [6].

Consequently, the capacity of a single specialized kidney progenitor cell to regenerate the complex structure of the renal nephron for kidney recovery is limited despite many research efforts focusing on the introduction of specific kidney progenitor cells such as metanephric mesenchyme (MM) and ureteric epithelium (UE). In addition, the reciprocal interaction between MM and UE is a critical process during kidney development, and their combined utilization represents a groundbreaking approach to kidney regeneration [5, 6]. In this content, intermediate mesoderm (IM) cells emerge as promising candidates with the ability to differentiate into multiple types of kidney progenitor cells. IM cells can be derived from human pluripotent stem cells (hf) with a serial addition of differentiation factors [7, 8]. IM cells have demonstrated multipotency by differentiating into two distinct kidney progenitor cells, namely MM and UE [8, 9]. By controlling IM differentiation at kidney injury sites and harnessing the reciprocal interaction between differentiated kidney progenitor cells, complete kidney tissue regeneration could be achieved.

To provide mechanical stability and support cell migration and signal induction with bioactive components for tissue regeneration and engineering, various types of scaffolds have been utilized [10–15]. Among these, biodegradable polymer-based scaffolds have been an attractive choice due to their ease of manipulation, controllable mechanical properties, and the absence of the need for additional processes to remove the scaffolds. These advantages have made biodegradable polymers widely used in various regenerative medicine applications, including bone regeneration, wound healing, nerve conduits, and cartilage repair [16–19]. One of these biodegradable polymers, poly(lactic-*co*-glycolic) acid (PLGA), has been particularly useful due to its appropriate mechanical, chemical, and biological properties. The formation of porous structures through ice particle leaching has played a crucial role in tissue regeneration by facilitating interactions with the surrounding environment and the sustained release of components [20–22]. However, it is essential to address the issue of inflammation and cellular necrosis in surrounding tissues caused by acidic byproducts resulting from PLGA hydrolysis. To address these concerns, we previously introduced ricinoleic acid-grafted magnesium hydroxide (Mg(OH)_2_-RA; M), which has pH-neutralizing effects, and further modified the extracellular matrix (ECM; E) to enhance biocompatibility within the PLGA (P) scaffolds for kidney tissue regeneration [23–27]. As expected, magnesium hydroxide exhibited antacid activity by combining with acidic byproducts of PLGA through ionized hydroxide. Furthermore, the incorporation of ECM enhanced the biocompatibility of porous PLGA by mimicking the complex microenvironments of the kidney. Based on PME scaffolds, nitric oxide (NO)-releasing material was applied to facilitate angiogenic activities in an optimum condition. NO is synthesized from nitric oxide synthase and plays a role in activating cell proliferation and regulating immune responses for angiogenesis [28–30]. Due to the limitations associated with NO-releasing biomolecules, including rapid thermal decomposition and limited exposure area [29, 31, 32], we successfully incorporated continuous NO-releasing alpha lipoic acid-conjugated zinc oxide (ZnO-ALA; Z) into PME scaffolds. Zinc oxide (ZnO) particles, as a NO-releasing metal oxide, reacted with innate glutathione peroxidase and glycosidase to allow the decomposition of donors and the release of NO under physiological conditions. ALA enabled continuous NO release by reacting with glutathione (GSH) and s-nitroso-N-acetylpenicillamine (SNAP) in bodily fluids, enhancing the dispersity of ZnO in hydrophobic conditions for sustained release from porous PLGA-based scaffolds [33, 34].

To enhance regeneration-related properties and restore kidney function, various bioactive materials have been applied in kidney injury models [35–38]. Extracellular vesicles (EVs) are a diverse group of lipid membrane-bound vesicles released by various cell types to facilitate intercellular communication [39–44]. EVs carry characteristics of their parent cells, and, notably, EVs derived from stem cells exhibit stem cell-like properties, making them valuable tools in regenerative medicine [45–48]. Furthermore, various strategies have been developed to modify parent cells, leading to engineered EVs with enhanced functionalities and desired properties [49–51]. Numerous cell preconditioning methods have been explored to control the bioactivities of extracellular vesicles released by target cells. These methods include manipulating culture conditions through three-dimensional culture and media composition adjustments [52], inducting pro-inflammatory cytokines to release immunomodulatory factors that maintain cellular homeostasis [53], and treating cells with bioactive molecules to modulate their activity [54]. A critical consideration is the isolation of EVs that fully represent the characteristics of parent cells and are free from impurities originating from cell culture media. To achieve this, various strategies have been employed, including the use of xeno-free, chemically defined, and human blood-derived alternatives to culture target cells for EV isolation, effectively removing animal-derived components while preserving cell proliferation and properties [55]. For example, we previously recommended the use of serum-free, chemically defined media, such as CellCor^™^ CD MSC (CDM), to isolate MSC-derived EV with high production yield and purity [56].

Moreover, a relatively large number of EVs could be isolated from healthy MSCs that were free from animal-derived proteins and other impurities while maintaining an excellent proliferation rate. Although several studies have highlighted the potential of engineered EVs in CKD treatments, the selection of appropriate cell types and EVs to modulate cellular activities at injury sites is crucial for tissue regeneration.

**Fig. 1:**
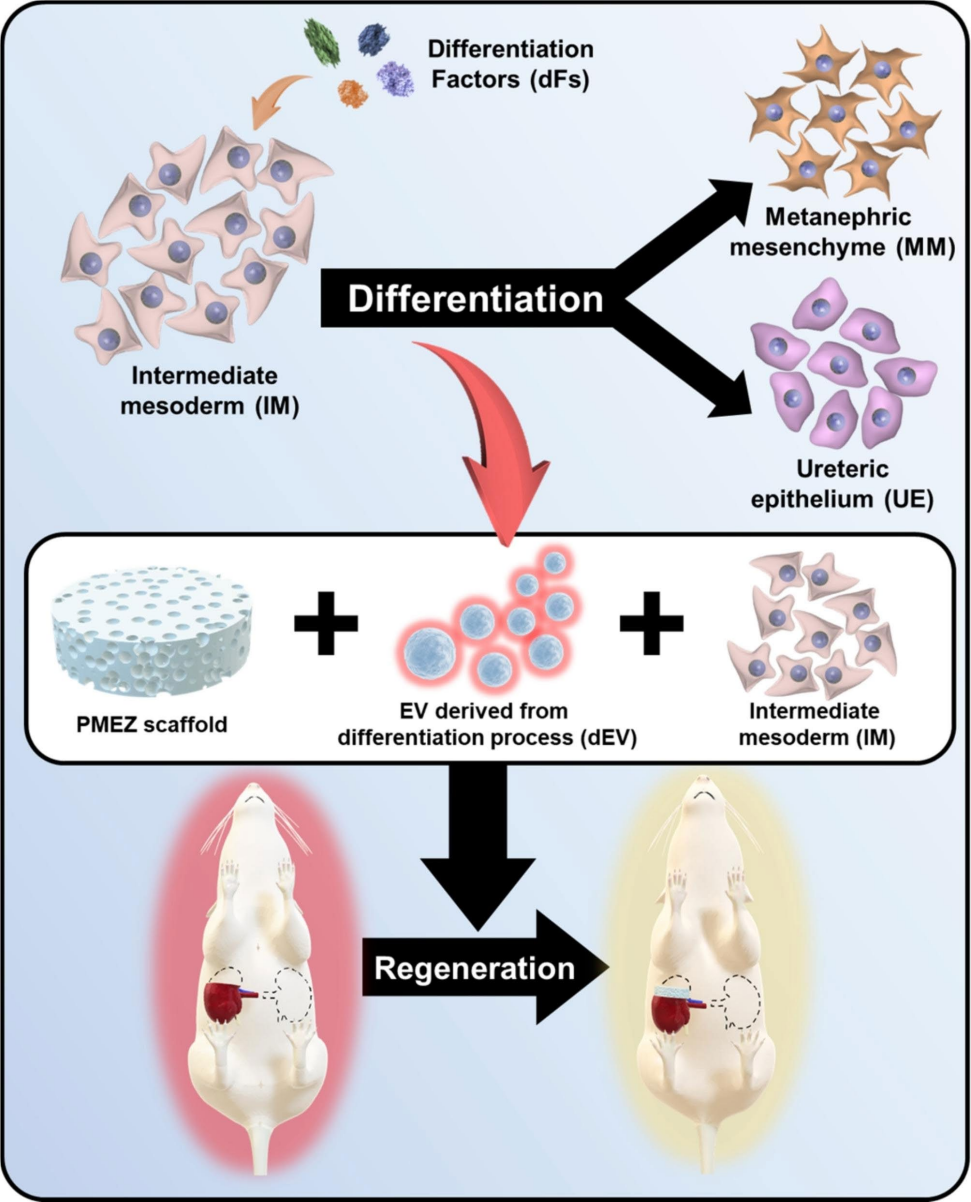
Schematic illustration of kidney tissue regeneration with bioactive PMEZ/dEV/IM scaffolds

In this study, we modified porous PMEZ scaffolds embedded with ricinoleic acid-grafted magnesium hydroxide (MH-RA; M) to enhance MH’s pH-neutralization ability, extracellular matrix (ECM; E) to mimic kidney tissue environment, and alpha lipoic acid-conjugated zinc oxide nanoparticles (ZnO-ALA; Z) to promote angiogenic properties with continuous dual NO release in kidney tissues based on the PLGA (P) platform. Additionally, human pluripotent stem cell-derived IM was introduced to regenerate the entire nephron structure by differentiating IM into various types of kidney progenitor cells with the addition of differentiating extracellular vesicles (dEVs). The effectiveness of dEV-mediated IM differentiation was confirmed in a three-dimensional scaffold system in vitro, and structural and functional kidney regeneration was demonstrated in a 3/4 nephrectomy nude mouse model, simulating kidney damage while eliminating immune responses from human-derived IM cells (Fig. 1).

## Materials and methods

### Materials

Poly(D,L-lactic-*co*-glycolic) acid) (PLGA, LA/GA = 50:50, MW 110,000) was obtained from Evonik Ind. (Essen, Germany). Magnesium hydroxide (Mg(OH)_2_, MH; M), Zinc Oxide (ZnO; Z), retinoic acid and heparin sodium salt were purchased from Sigma-Aldrich (St. Louis, MO, USA). Ricinoleic acid and DL-α-lipoic Acid (ALA) were purchased from TCI product (Tokyo, Japan). ELISA kits (R&D Systems, Minneapolis, MN, USA) were purchased form R&D Systems. Recombinant human fibroblast growth factor 9 (FGF9) and bone morphogenetic protein 7 (BMP7) were purchased from Peprotech (Rocky Hill, USA).

### Cell culture and differentiation

Human pluripotent stem cell derived intermediate mesoderm (IM) was manufactured by Prof. Dong Ryul Lee (CHA Univ., Gyeonggi, Korea) [57] and cultured using CellCor™ CD MSC media (CDM; Xcell Therapeutics, Seoul, Korea) containing 1% antibiotic-antimycotic solution for serum-free condition. The day before starting differentiation, the cells were seeded at 15,000 cells/cm^2^ on a plate. After 3–5 days, the cells were exposed to FGF9 (200 ng/ml), BMP7 (50 ng/ml), retinoic acid (RA; 30 ng/ml), and heparin (1 µg/ml) for 12 days as described in the previous report [8]. To differentiate IM using EV derived from the differentiation process (dEV), we determined the amount of EVs based on the concentration of FGF9 and BMP7 encapsulated within the EVs (200 ng/mL and 50 ng/mL, respectively). The conditioned media were obtained in every two days during replacing media.

### Isolation and characterizations of extracellular vesicles from differentiating IM (dEV)

To prepare for EV isolation, the culture media (CM) were collected in every two days during changing media for 12 days. The collected CM were subjected to centrifugation at 1,300 rpm for 3 min to remove larger particles (such as cells, cell debris, microvesicles, apoptotic bodies, etc.) than EVs. To isolate EV, 0.22 μm filtered CM was applied with a tangential flow filtration system (KR2i TFF; Repligen, Waltham, MA, USA) using 500 kDa molecular weight cut-off filter. The isolated EV was concentrated using the Amicon ultra-15 centrifugal filter (Merck Millipore, Billerica, MA, USA). The size distribution and the quantity of EVs were determined with MONO ZetaView® with 488 nm scatter mode (PMX-120, Particle Metrix, Meerbusch, Germany). The EVs were diluted with filtered phosphate-buffered saline (PBS) solution (WelGENE Inc., Daegu, Korea) to 10^7^–10^8^ particles/ml before the evaluation. The measurement parameters were tuned with a sensitivity 75, shutter 100, minimum trace length 15, and cell temperature at 25 °C for accurate analysis. The morphology of EV was identified using transmission electron microscopy (TEM; Hitachi, H-7600, 80 kV, Japan). For a negative staining procedure, the EV solution was dried on a 150-meshed formvar/carbon supported copper grid (FCF150-CU, Electron Microscopy Sciences, USA) and stained with UA-Zero (Agar Scientific, Stansted, UK) solution.

### Fabrication and characterizations of bioactive scaffolds

The PMEZ scaffold integrated with ricinoleic acid-grafted magnesium hydroxide (MH-RA), ADM, and DL-α-lipoic acid-conjugated zinc oxide (ZnO-ALA) based on PLGA were fabricated using the ice particle leaching technique. The PLGA (0.25 g), MH-RA (15 wt%), ADM (20 wt%) and ZnO-ALA (10 wt%) were dissolved and mixed in dichloromethane (DCM; 1.9 ml) to manufacture PMEZ scaffolds. After freeze drying process to eliminate volatile organic components, we loaded 3 × 10^6^ IM cells onto the hydrated PMEZ scaffold and stabilized for 24 h and incorporated 4.5 × 10^8^ particles of dEV onto the PMEZ/IM scaffold. The porosity of the scaffold was identified using field emission-scanning electron microscopy (FE-SEM, SIGMA, Carl Zeiss). Inorganic components within the scaffold were analyzed using thermogravimetric analysis (TGA, PerkinElmer, Waltham, MA). The mechanical properties of the scaffold were evaluated using the Universal testing machine (UTM) on an Instron 4464 instrument (Instron, Norwood, MA). To confirm the distribution of IM within the scaffold, IM was stained with Hoechst33342 (Invitrogen, CA, USA). The stained components were individually loaded onto the scaffold and observed using a confocal laser microscopy (LSM880, Carl Zeiss, GE). The nitric oxide (NO) concentrations were measured using slightly modifying electrochemical microsensors described in previous report by Moon et al. [58].

### Western blot analysis

Cells were resuspended in RIPA buffer containing protease inhibitors. For the comparison of two types of EVs, an equal amount of EVs (1 × 10^9^ particles) and an equivalent number of cells as EV negative controls were loaded to 10% sodium dodecyl sulfate-polyacrylamide gel electrophoresis (SDS-PAGE). Subsequently, proteins were transferred onto nitrocellulose (NC) membranes for analysis. Primary antibodies used for immunoblotting were selected based on MISEV guidelines for EV validation, including CD63 (Abcam, MA, USA), TSG101, and Apo-A1 (Santa Cruz Biotechnology, CA, USA). Additionally, PAX2, SIX2 (Abcam, MA, USA) and WT1, GAPDH (Santa Cruz Biotechnology, CA, USA) were chosen to confirm the differentiation of human IM. The HRP-linked secondary antibody (Cell Signaling Technology, MA, USA) was used for the detection of the marker intensity. The membrane was exposed with an enhanced chemiluminescence solution (ECL; GE Healthcare, WI, USA) and visualized using ChemiDoc™ XRS + with ImageLab software (Bio-Rad, CA, USA).

### Enzyme-linked immunosorbent assay (ELISA) assay

The differentiation factors (FGF9 and BMP7) in EVs were verified using the Quantikine™ ELISA kit (R&D Systems, MN, USA). The same quantity of EVs (1 × 10^7^ particles/well) was performed, and the process was carried out in accordance with the manufacturer’s instructions. The concentration of the factor was determined with the absorbance at 450 nm measurement using a microplate reader (Molecular Devices, CA, USA).

### Cell viability assay

The cell viability was obtained using the Cell Counting Kit-8 (CCK-8; Dojindo, Kumamoto, Japan). To determine relative cell viability, the CCK-8 test was performed with the manufacturer’s instructions. The absorbance was measured at a wavelength of 450 nm using a microplate reader (Molecular Devices, CA, USA) to evaluate cell viability.

### Cell differentiation assessment using three-dimensional (3D) scaffolds

To evaluate cell differentiation with 3D scaffolds, IM was seeded at a density of 15,000 cells/cm² on 6-well plates. Subsequently, a co-culture system was established using trans-well inserts (36,206, SPLInsert™, SPL, Korea), and biodegradable scaffolds were added on the upper of the insert. After 3–5 days of cell seeding, the dEV was added for differentiation. The culture media with dEV were changed every two days. The differentiation process was performed for 12 days as described in a previous report [8].

### Real-time quantitative PCR (RT-qPCR) analysis

The AccuPrep® Universal RNA Extraction Kit (Bioneer, Daejeon, Korea) was used to extract the total cellular RNA. Reverse transcription for converting extracted RNA to cDNA was carried out using the PrimeScript™ RT reagent kit (Takara, Shiga, Japan). For RT-qPCR, a combination of SYBR green PCR reagents (Applied Biosystems, CA, USA) was utilized. QuantStudio 3 (Applied Biosystems, CA, USA) was used to proceed the reactions with the primers. To quantification of the data, the 2^−ΔΔCt^ method was applied with 18s rRNA as a reference. Primers for in vitro analyzes were listed in Table S1.

### Design for in vivo model

All in vivo experiment protocols were approved by the institutional animal ethics committee of Yeungnam University, College of Medicine (YUMC-AEC2022-023). Six-week-old female nude mice were purchased from Jung Ang Lab Animal Inc (Seoul, Korea) and randomly divided into 5 groups (Native, PMEZ, PMEZ/dEV, PMEZ/IM, and PMEZ/dEV/IM). The scaffolds were implanted into 3/4 nephrectomy mice models after whole nephrectomy of left kidney and partial nephrectomy of right one. Mice were sacrificed at 2 and 8 weeks after implantation of scaffolds. Animals were anesthetized with 16 mg/kg of rompun and 0.04 mg/kg zoletil by intramuscular injection.

### Histological analysis

All collected kidney tissues were fixed in 10% formalin and embedded in paraffin. Tissue sections were cut into 4 μm thickness and applied on coated slide glasses. We proceeded Hematoxylin and Eosin (H&E; Abcam, MA, USA) staining and Massons’s Trichrome (MT; Abcam, MA, USA) staining for general histology and fibrosis, respectively. Moreover, periodic acid Schiff (Sigma-Aldrich) was stained for glomerulus visualization using commercial kits following the manufacturer’s instruction. Slides were examined under light microscopy. For immunohistochemistry, slides were deparaffinized and hydrated by xylene and ethanol. Citrated buffer was used for antigen retrieval prior to blocking step with 5% BSA solution. Primary antibodies (1:100) were applied for 18 h at 4 ˚C. FITC conjugated secondary antibody were applied for 2 h at room temperature. The slides were mounted with DAPI staining medium (Vector Laboratories, Burlingame, CA, USA) and examined under fluorescence microscopy.

### Real-time quantitative PCR (RT-qPCR) of in vivo samples

Maxwell® RSC simply RNA cell kit was used to isolate RNA from kidney tissues by operating Maxwell™ 16 instrument (Promega Corporation, Madison, WI, USA). One ug of isolated RNA was used to synthesize cDNA with GoScript TM Reverse Tanscription Mix (Promega Corporation, Madison, WI, USA) according to the product protocols. Real-time PCR was performed in StepOnePlus™ Real-Time PCR System (Applied Biosystems® Inc., Foster City, CA, USA) by using LUNA NEB SYBR Green Master Mix (NEB, MA, USA). Primers for in vivo analyzes were listed in Table S2.

### Statistical analysis

The program GraphPad Prism 9 (GraphPad program, CA, USA) was used for the statistical analyses. Unpaired *t-*tests or one-way analysis of variance (ANOVA), followed by Tukey’s multiple comparison post-test, were used to analyze group differences. *P* values less than 0.05 were used to show statistical significance, with levels marked as (*p < 0.05; ** *p* < 0.01; *** *p* < 0.001; **** *p* < 0.0001).

## Results

### Physicochemical properties of biodegradable porous scaffolds

**Fig. 2:**
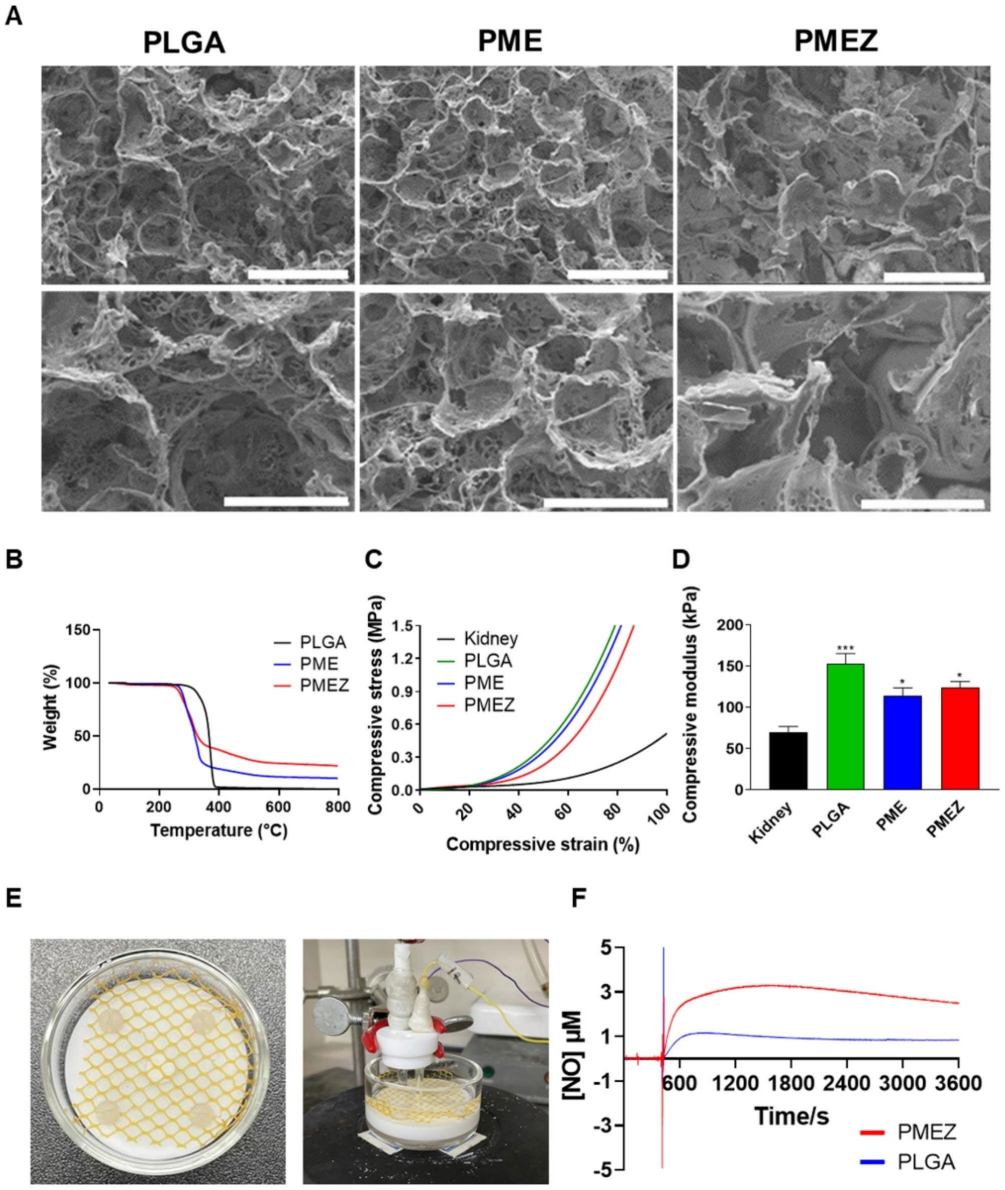
Characterization of biodegradable porous scaffolds. (**A**) Representative SEM images of the PLGA, PME, and PMEZ scaffolds (scale bars, up: 200 μm, and bottom: 100 μm). (**B**) Thermogravimetric analysis (TGA) thermograms of the PLGA, PME, and PMEZ scaffolds. (**C**) Compressive stress-strain curve and (**D**) compressive modulus at 5 to 10% of stress-strain curve. (**E**) Representative confocal images showing distribution of DiO-labeled EV, and Hoechst-labeled IM in the PMEZ scaffolds (Scale bar equals to 20 μm). (**F**) Nitric oxide release profiles using the electrochemical microsensor. (Values are presented as mean ± SD (n = 3) and statistical significance was obtained with unpaired *t* tests or one-way analysis of variance (ANOVA) with Tukey’s multiple comparison post-test (*p < 0.05; **p < 0.01; ***p < 0.001; ****p < 0.0001))

The biodegradable porous PLGA matrix was selected as the platform for the scaffold, and it was functionalized with bioactive materials, as previously reported [24, 49, 59]. The scaffolds, featuring interconnected pores and high porosity, were created using the ice particle leaching method, offering advantages in terms of ease of manipulation and control over size. Similar porosities (100 ~ 200 μm) were achieved with the incorporation of functional materials, such as MH-RA (M), ECM (E), and ZnO-ALA (Z), in PLGA scaffolds, using the same ratio of ice particles to facilitate the migration and diffusion of bioactive components between the scaffolds and peripheral tissues (Fig. 2a). Distinct profiles of thermal decomposition were observed upon the addition of inorganic functional materials, MH-RA and ZnO-ALA, as shown in the thermogravimetric analysis. Both PME and PMEZ scaffolds exhibited an earlier onset of thermal decomposition compared to native PLGA scaffolds, owing to the relatively low proportion of pyrolyzable components resulting from the addition of bio-functional inorganic materials (Fig. 2b). The remaining weight increased with the addition of ECM, MH-RA, and ZnO-ALA after PLGA decomposition. The amount of incorporated ECM, MH-RA (15 wt%), and ZnO-ALA (10 wt%) were indirectly calculated based on the residual weight of the scaffolds. To analyze the mechanical properties of the scaffolds, compressive strain-stress curves were obtained using a Universal testing machine (Fig. 2c). The compressive modulus, calculated as the slope between 5 ~ 10% of the strain-stress curve, decreased with the addition of MH-RA and ECM. At the same time, it showed a negligible increase with the addition of ZnO-ALA, resulting in a modulus similar to that of the kidney when compared to bare PLGA scaffolds (Fig. 2d).

The release properties of nitric oxide (NO) were examined using electrochemical microsensors, following the method established by Moon et al. [58]. The electrochemical microsensor comprised a reference electrode and an NO detection component with the Ag/AgCl and Pt wire, respectively (Fig. 2e). By creating a reticular structure to immobilize the scaffolds, the No release profile was detected in the presence of NO donors, glutathione (GSH), and s-nitroso-N-acetylpenicillamine (SNAP), simulating condition in body fluids (Fig. 2f). The amounts of released NO were calculated using a standard curve relating current to the concentration of NO (Figure S1). When only GSH and SNAP were present as NO donors with PLGA, weak signals were detected due to the natural decomposition of NO donors. In contrast, significantly stronger signals were generated with PMEZ scaffolds. The continuous NO-releasing profile derived from ZO-ALA in the PMEZ scaffold was monitored, with relatively higher amounts of NO generated over time due to the sustained release of ZnO-ALA from PMEZ scaffolds.

### Characterization of EVs isolated from kidney differentiating intermediated mesoderm in chemically defined media (CDM)

**Fig. 3:**
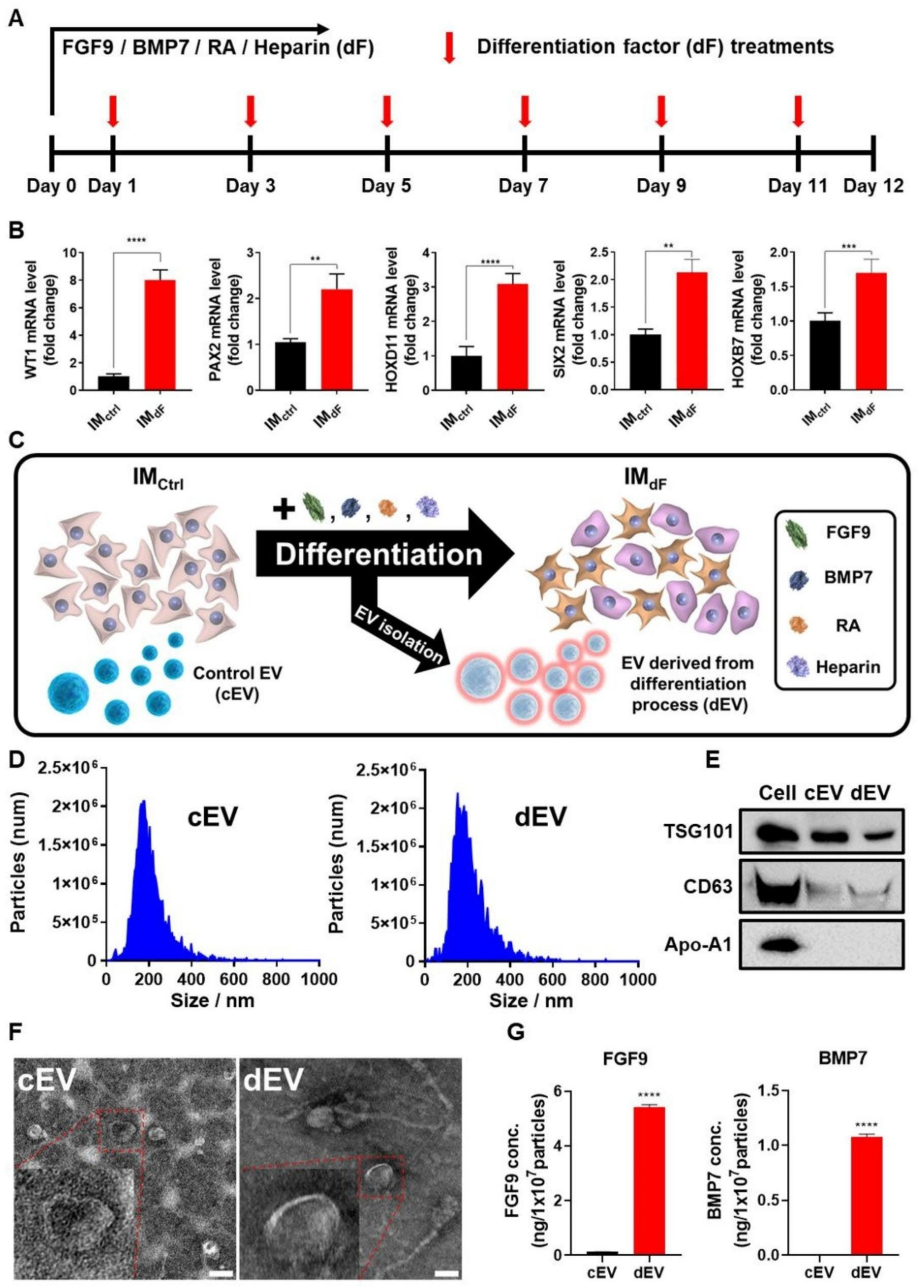
Characterization of renal differentiating extracellular vesicles. (**A**) Time schedules for renal differentiation and acquisition of differentiating EV (dEV). (**B**) The gene expression levels of renal differentiation factors in IM_ctrl_ and IM_dF_ by quantitative PCR with reverse transcription (RT-qPCR; n = 3). (**C**) The overview of isolations for two types of EVs (cEV and dEV). (**D**) Zetaview analysis for the number and size of total particles of cEV and dEV. (**E**) Western blot analysis of cEV and dEV for representative markers of EVs. (**F**) Assessment of EV morphologies by transmission electron microscopy (TEM). (Scale bars equal to 100 nm). (**G**) Quantification of differentiation factors (FGF9, and BMP7) in cEV and dEV using ELISA analysis. (Values are presented as mean ± SD (n = 3) and statistical significance was obtained with unpaired *t* tests or one-way analysis of variance (ANOVA) with Tukey’s multiple comparison post- test (*p < 0.05; **p < 0.01; ***p < 0.001; ****p < 0.0001))

To obtain differentiation factors incorporated EVs, EVs were isolated from intermediated mesoderm (IM) during kidney differentiation. Cells that have been preconditioned with specific factors can release EVs with same components and functions [49]. Human pluripotent stem cell (hPSC)-derived IM has been developed to differentiate into two types of cells for kidney construction, metanephric mesenchyme (MM) and ureteric epithelium (UE) with the aid of FGF9, BMP7, retinoic acid (RA), and heparin, over a period of twelve days in serum-enriched APEL media [8, 60–62]. In particular, in this study, to eliminate impurities and enhance EV functionalities, IM was cultured and differentiated under serum-free conditions using chemically defined media, CellCor™ CD MSC (CDM). To confirm renal differentiation of IM in CDM, IM was cultured and differentiated for twelve days under the same conditions as those used for differentiation in serum-enriched APEL media (Fig. 3a). Interestingly, cells differentiated using CDM exhibited morphological changes and expressed kidney differentiation-related markers in a similar pattern as when using serum-enriched APEL media (Figures S2a and b). PCR analysis demonstrated that representative renal (WT1, PAX2), MM (HOXD11 and SIX2), and UE (HOXB7) markers were expressed more in IM differentiated in CDM compared to IM, indicating successful differentiation of IM into renal progenitor cells in CDM (Fig. 3b). To verify the differentiation properties of EVs released during IM differentiation, conditioned media were collected every two days to isolate differentiating EVs (dEVs) during IM differentiation into MM and UE (Fig. 3c). After isolating dEV using the tangential flow filtration system, two types of EVs, cEVs and dEVs, were characterized following the guidance of the MISEV 2018 guidelines for EV characterization [39]. cEVs were isolated from IM to compare their differentiation properties with dEVs. No significant differences in the size and number of EVs were detected in both types of EVs (Fig. 3d). The presence of CD63 and TSG101, representative transmembrane and intracellular proteins found in EVs, was clearly detected in western blot analysis, while one of the major negative markers, apolipoprotein A1 (Apo-A1), was not expressed in either type of EV (Fig. 3e). Furthermore, double-layered spherical structures were observed using transmission electron microscopy (Fig. 3f). By isolating the EVs during IM differentiation into renal cells, two major renal differentiation-related factors, FGF9 and BMP7, were successfully incorporated in dEVs compared to cEVs (Fig. 3g). The significant difference in the amount of differentiation factors shown in the ELISA results is attributed to the added differentiation factors but also the factors released by the renal progenitor cells during differentiation [7, 45, 63].

### Renal differentiation activities of dEV

**Fig. 4:**
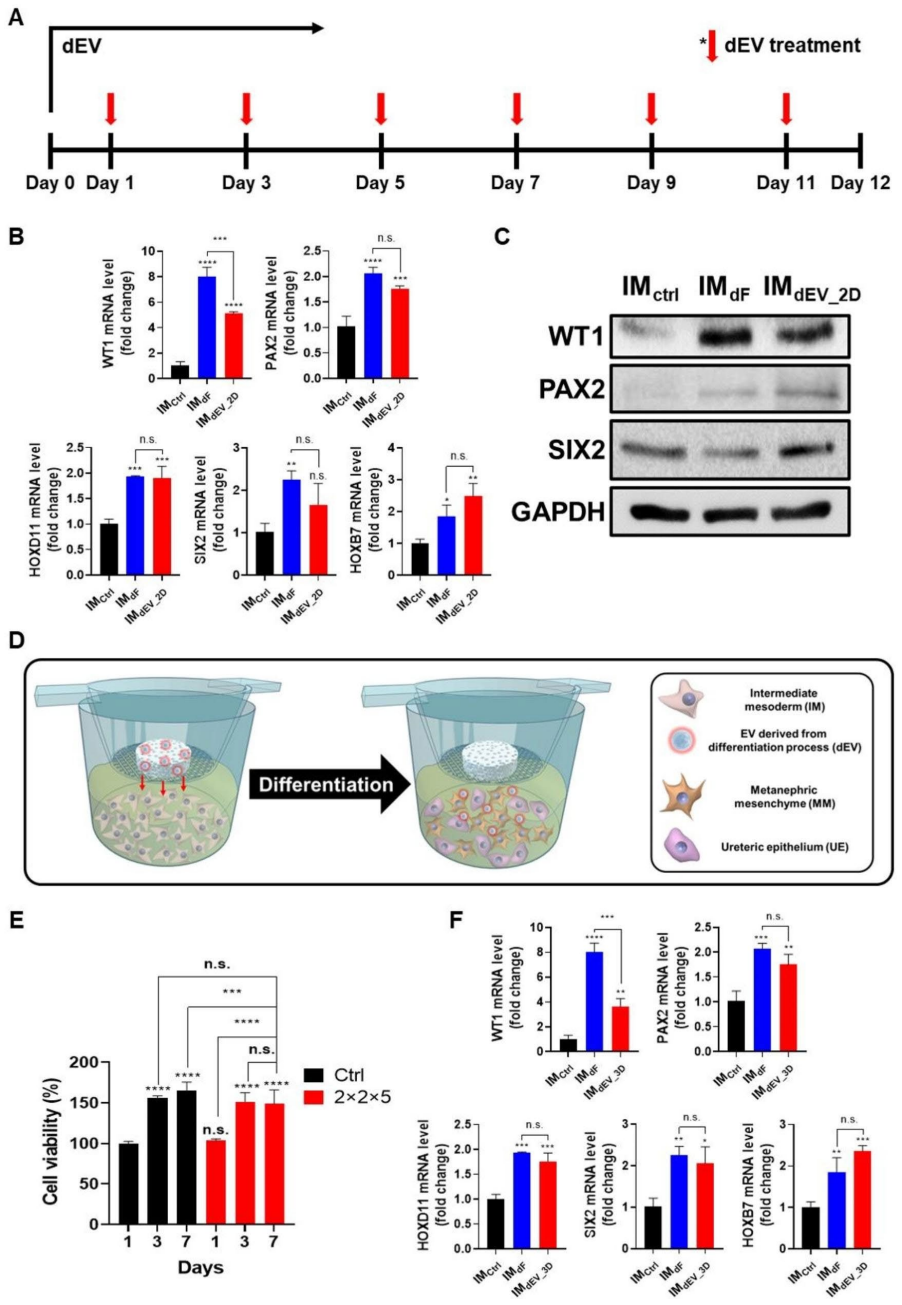
IM differentiation into kidney progenitor cells in 2D and 3D culture conditions in vitro. (**A**) Time schedules for renal differentiation by differentiating EV (dEV) in 2-dimensional (2D) condition. (**B**) The gene expression levels of renal differentiation factors in IM_ctrl_ and IM_dEV−2D_ by RT-qPCR (n = 3). (**C**) Western blot analysis of representative differentiation markers for IM_ctrl_, IM_dF_ and IM_dEV−2D_. (**D**) The schematic image of IM differentiation into renal progenitor cells in 3-dimensional condition using transwell culture system with PMEZ/dEV scaffolds. (**E**) The proliferation of IM with or without PMEZ scaffold on transwell system for 7 days (n = 3). (**F**) The gene expression levels of renal differentiation factors in IM_ctrl_ and PMEZ/dEV/IM (IM_dEV−3D_) by RT-qPCR (n = 3). (Values are presented as mean ± SD (n = 3) and statistical significance was obtained with unpaired *t* tests or one-way analysis of variance (ANOVA) with Tukey’s multiple comparison post-test (*p < 0.05; **p < 0.01; ***p < 0.001; ****p < 0.0001))

Our study is based on the evidence that IM differentiates into renal progenitor cells over a twelve-day period when exposed to differentiation factors. We treated 4.5 × 10^8^ dEV particles every two days in the IM culture in CDM, replacing the differentiation factors (Fig. 4a). We determined the number of particles by assessing the number of dEVs containing similar amounts of FGF9 and BMP7, which are major differentiation factors required for IM differentiation into renal progenitor cells. The analysis of gene expression levels upon completion of differentiation revealed that renal differentiation was achieved with dEV without the need for additional differentiating factors (Fig. 4b). When we examined the gene expression levels of representative renal (WT1 and PAX2), MM (HOXD11 and SIX2), and UE (HOXB7) markers, we observed elevated levels after introducing dEV (IM_dEV-2D_) compared to IM (IM_Ctrl_). On the other hand, LHX1, which is typically expressed in undifferentiated IM cells, displayed a significant decrease in expression in the IM_dEV-2D_ group (Figure S4a). In addition, we assessed the renal differentiation properties of dEVs in a three-dimensional environment by exposing inner medullary cells to dEV-incorporated PMEZ scaffolds (PMEZ/dEV). IM cells incubated with dEV (IM_dEV-2D_) exhibited representative renal differentiation markers expression in a similar trend to IM differentiated with differentiation factors (IM_dF_, Fig. 4c). With verifying the well-organized scaffolds of dEV and IM using confocal microscopy (Figure S3), IM cell was exposed to dEV incorporated PMEZ scaffolds (PMEZ/dEV) to further investigate renal differentiation property of dEV in the three-dimensional environment.

To further investigate the renal differentiation property of dEV in the three-dimensional environment, IM cells were exposed to dEV-incorporated PMEZ scaffolds (PMEZ/dEV). To ensure that IM cells were not lost during the process, we conducted PMEZ/dEV-mediated differentiation using an insert system-based indirect method as described in Fig. 4d. After confirming the non-cytotoxicity of PMEZ/dEV to IM cells, we investigated IM differentiation by evaluating gene expression levels of renal markers twelve days after cell incubation in PMEZ/dEV-inserted plates (Fig. 4e). Consistent with our findings in two-dimensional conditions, IM cells successfully differentiated when exposed to dEV incorporated into PMEZ scaffolds (IM_dEV-3D_). Specifically, gene expression levels of renal, MM, and UE markers increased upon incubation with dEV-incorporated PMEZ scaffolds, compared to IM (Fig. 4f), whereas LHX decreased as IM differentiation progressed (Figure S4b).

### In vivo evaluation of PMEZ/dEV/IM for renal differentiation and regeneration-related bioactivities

**Fig. 5:**
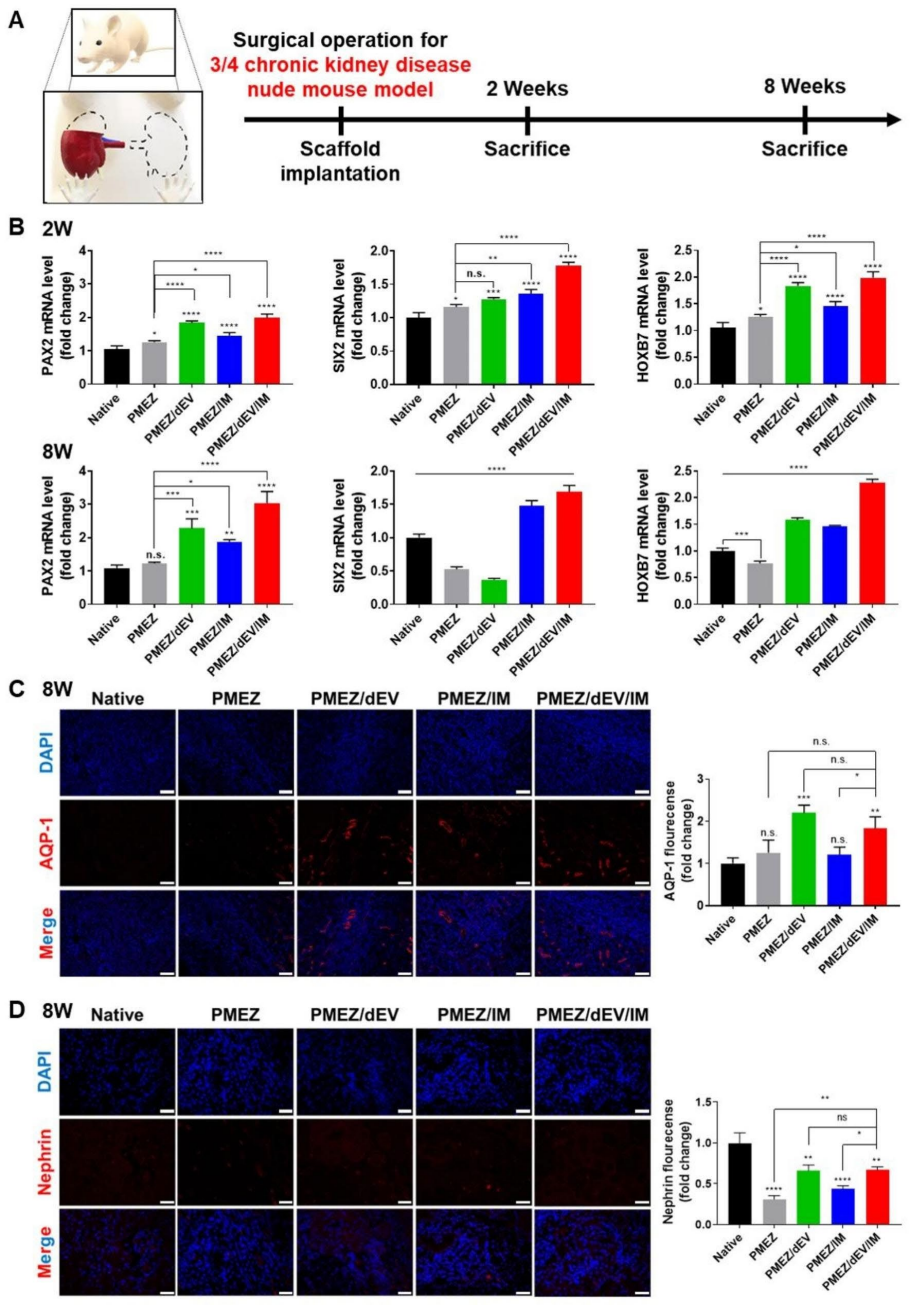
In vivo evaluations for renal differentiation-related bioactivities. (**A**) Time schedules for scaffold implantations and in vivo analysis for 3/4 nephrectomy mice models (**B**) The gene expression levels of differentiation markers (PAX2, SIX2, and HOXB7) at 2 and 8 weeks after implantations (n = 3). (**C**) The fluorescence-based immunohistochemistry and quantitative analysis of AQP-1 expression for Native, PMEZ, PMEZ/dEV, PMEZ/IM, and PMEZ/dEV/IM implantations at 8 weeks. (**D**) The fluorescence-based immunohistochemistry and quantitative analysis of Nephrin expression of Native, PMEZ, PMEZ/dEV, PMEZ/IM, and PMEZ/dEV/IM implantations at 8 weeks. Scale bars equal to 100 μm (Values are presented as mean ± SD (n = 3) and statistical significance was obtained with unpaired *t* tests or one-way analysis of variance (ANOVA) with Tukey’s multiple comparison post-test (*p < 0.05; **p < 0.01; ***p < 0.001; ****p < 0.0001))

Four types of scaffolds, PMEZ, PMEZ/dEV, PMEZ/IM, and PMEZ/dEV/IM, were implanted into nephrectomy mouse models. In the case of IM-introduced scaffolds, the scaffolds were implanted directly after seeding IM cells to minimize the possibility of IM cells being left out of the scaffolds. To avoid immune rejection caused by human-derived IM cells, a 3/4 nephrectomy nude mouse model was used as the CKD-simulated animal defect model in this study. All parameters were evaluated at 2 and 8 weeks after scaffold implantations in the injured kidney tissues (Fig. 5a). The mRNA expression levels of representative kidney, MM, and UE markers, PAX2, SIX2, and HOXB7, respectively, were evaluated to demonstrate the renal differentiation capacities of the scaffolds. PMEZ/dEV and PMEZ/IM scaffolds showed slightly higher signals compared to PMEZ scaffolds. Furthermore, these expression levels significantly increased in PMEZ/dEV/IM scaffolds (Fig. 5b). PMEZ scaffolds with dEV or IM alone undergo differentiation and regeneration to some extent due to cells and various factors in the remaining tissues in the 3/4 nephrectomy model. However, more complete tissue regeneration through differentiation can be expected when cells and differentiation factors are introduced together. To further investigate renal differentiation-mediated kidney regeneration, detailed staining was performed for representative markers of tubules and podocytes at 2 and 8 weeks after scaffold implantation (Fig. 5c and d and Figure S5). The populations of both aquaporin 1 (AQP1) and nephrin were enhanced in the PMEZ/dEV and PMEZ/IM groups and significantly upregulated in the PMEZ/dEV/IM group.

Kidney fibrosis is the common pathological characteristic that results in CKD. It occurs due to the deregulation of wound healing and the accumulation of excessive extracellular matrix proteins [64, 65].

**Fig. 6:**
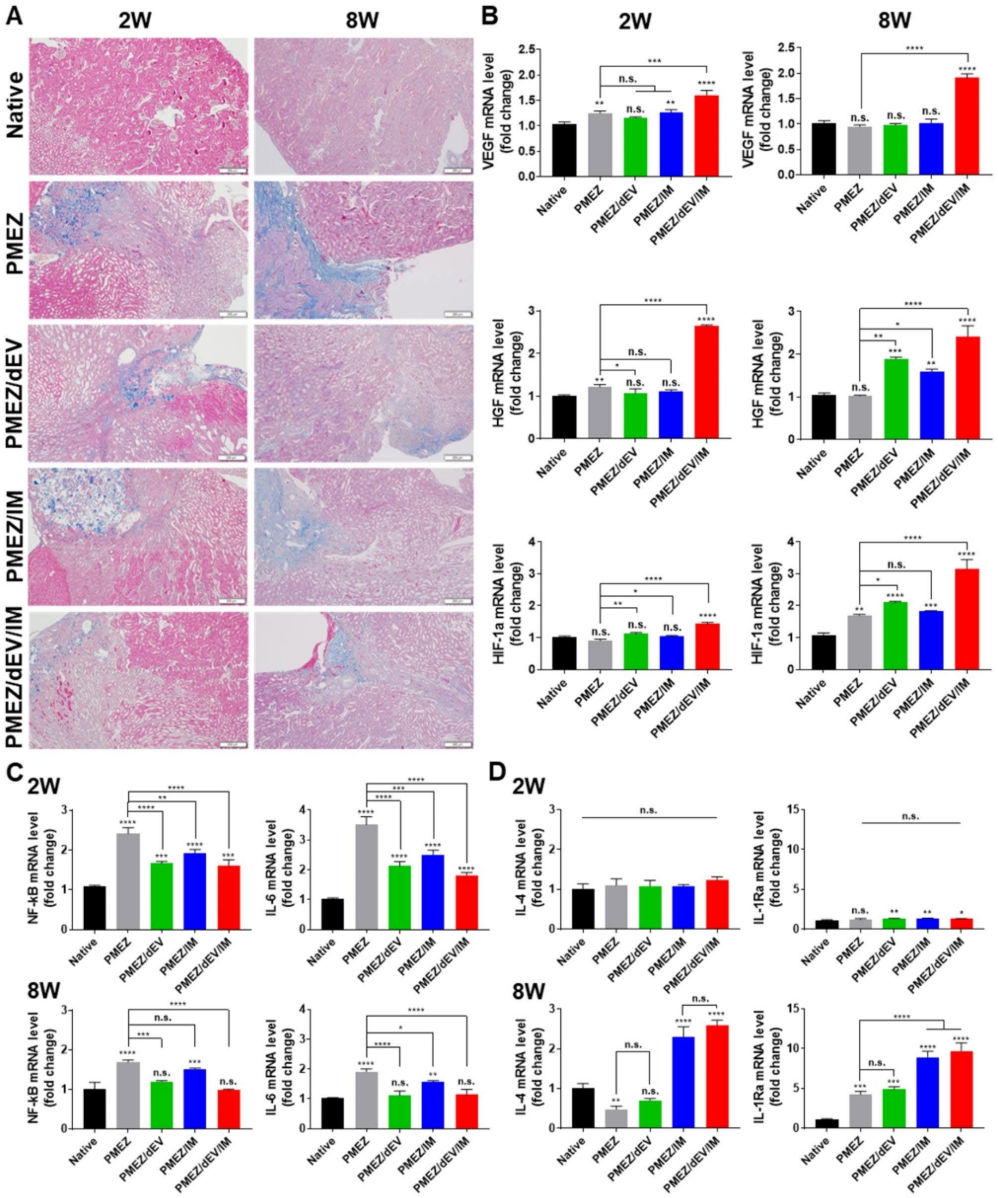
In vivo evaluations for regeneration-related bioactivities of the scaffolds. (**A**) Representative PAS staining of scaffold region for assessment of fibrosis at 2 and 8 weeks after implantations (Scale bars equal to 200 μm). (**B**) The gene expression levels of angiogenic markers (VEGF, HGF, and HIP-1α) at 2 and 8 weeks after implantations (n = 3). (**C**) The gene expression levels of pro-inflammatory factors (NF-kB and IL-6) and (**D**) anti-inflammatory factors (IL-4, IL-1Ra) at 2 and 8 weeks after implantations (n = 3). (Values are presented as mean ± SD (n = 3) and statistical significance was obtained with unpaired *t* tests or one-way analysis of variance (ANOVA) with Tukey’s multiple comparison post-test (*p < 0.05; **p < 0.01; ***p < 0.001; ****p < 0.0001))

Particularly in nephrectomy models, ECM proteins are successively accumulated in the peripheral tissues with severe injuries, leading to tissue fibrosis and renal dysfunction. To assess the fibrotic tissue recovery ability of the scaffolds, differences in collagen formation were compared in the surrounding tissues of various types of scaffold implantations using Masson’s Trichrome analysis (Fig. 6a). The presence of PMEZ/dEV/IM scaffolds resulted in a significant reduction in fibrotic tissue compared to the PMEZ scaffold, indicating strong antifibrotic effects when using a combination of bio-functional scaffolds with dEV and IM. The expression levels of three types of angiogenesis-related genes were upregulated in the PMEZ groups, with sustained release of ZnO-ALA, and were maximized with the incorporation of dEV and IM (Fig. 6b). While the anti-inflammatory effect of ricinoleic acid-grafted Mg(OH)_2_ has been demonstrated, PMEZ scaffolds have limitations in reducing inflammation in severely injured nephrectomy tissue. However, pro-inflammatory cytokines, such as nuclear factor kappa B (NF-kB) and interleukin-6 (IL-6), decreased with the incorporation of dEV and IM. As a result, their expression levels were similar to those of the native group at eight weeks (Fig. 6c and S6a). Conversely, anti-inflammatory cytokines, IL-4 and IL-1Ra, increased in these groups at eight weeks due to the inhibition of pro-inflammatory cytokine release through the bioactivities of PMEZ/dEV with IM (Fig. 6d and S6b).

### In vivo regeneration and functional restoration of kidney tissues with PMEZ/dEV/IM scaffolds

**Fig. 7:**
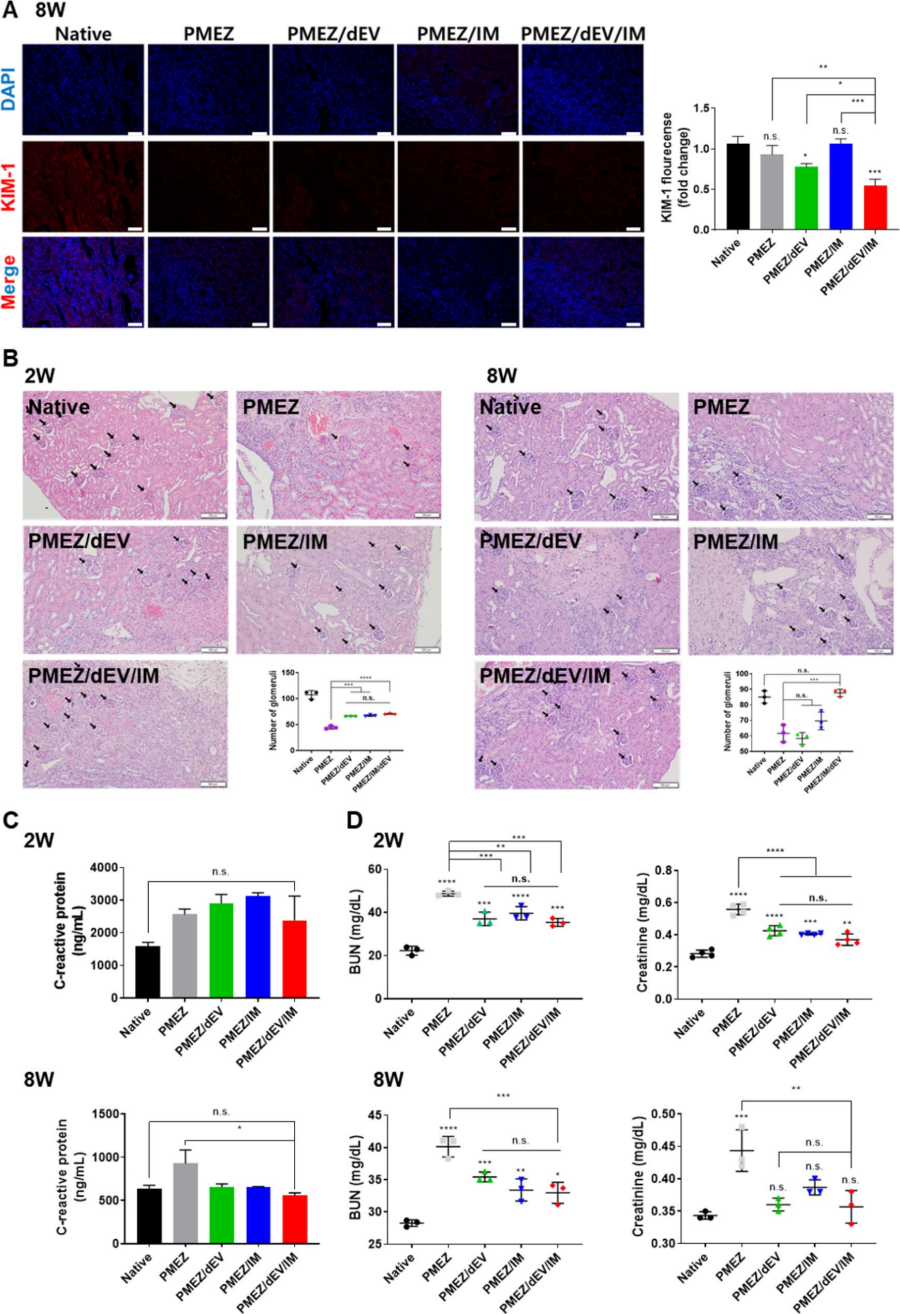
In vivo evaluations for kidney tissue regeneration and kidney function restoration properties of the scaffolds. (**A**) The fluorescence-based immunohistochemistry and quantitative analysis of AQP-1 expression for Native, PMEZ, PMEZ/dEV, PMEZ/IM, and PMEZ/dEV/IM 8 weeks after implantations. (Scale bars equal to 100 μm). (**B**) Representative H&E staining of scaffold region and quantitative analysis for assessment of regenerated glomeruli at 2 and 8 weeks after implantations (Scale bars equal to 100 μm). (**C**) Mouse body weight and the level of blood urea nitrogen (BUN) and creatinine in the serum at 2 and 8 weeks after implantations (n = 3). (**D**) CRP level at 2 and 8 weeks after implantations (n = 3). (Values are presented as mean ± SD (n = 3) and statistical significance was obtained with unpaired *t* tests or one-way analysis of variance (ANOVA) with Tukey’s multiple comparison post-test (*p < 0.05; **p < 0.01; ***p < 0.001; ****p < 0.0001))

To confirm kidney tissue recovery with the implantation of scaffolds in the 3/4 nephrectomy model, the expression of the representative biomarker for renal proximal tubule injury, kidney injury molecule 1 (Kim-1), was evaluated using immunocytochemical analysis (Fig. 7a and S7). As expected, the intensity of Kim-1 was downregulated with the addition of dEV and showed the lowest expression level with the synergistic effect of dEV and IM. Next, the number of glomeruli was counted in the peripheral tissue of scaffold implantations with histological staining. Glomeruli are considered incapable of regeneration in adult tissues because the podocyte, a critical constituent of the glomerular filtration barrier, cannot proliferate again once it is damaged beyond a certain threshold. One possibility for reviving podocytes is the introduction of renal progenitor cells [66].

Compared to the glomeruli regeneration observed two weeks after scaffold implantation, the number of glomeruli increased to levels similar to that of the healthy mouse model (Native group) when PMEZ scaffolds were incorporated with dEV and IM cells for eight weeks (Fig. 7b). The increased expression levels of kidney development-related factors, Pax2, SIX2, and HOXB7, as described previously, suggest that the incorporated IM cells with dEV in PMEZ scaffolds may differentiate into kidney progenitor cells, facilitating glomeruli regeneration with various physiologically active components in the scaffolds. However, further studies are needed to elucidate the precise mechanisms. The level of C-reactive protein (CRP), representing acute inflammation due to severe infection, injury, and/or chronic disease, was not completely inhibited in all types of groups but significantly recovered in the PMEZ/dEV/IM group at eight weeks (Fig. 7c). Finally, the restoration of kidney functions was comparatively analyzed using biochemical evaluation of levels of serum blood urea nitrogen (BUN) and creatinine (Fig. 7d). Within two weeks after scaffold implantation, the levels of BUN and creatinine started to decrease. Finally, the creatinine level dropped to a level similar to that of the native group at eight weeks.

## Discussion

As the number of patients suffering from chronic kidney disease (CKD) continues to rise, current therapies for end-stage kidney disease (ESKD) have primarily focused on two representative approaches, dialysis and kidney transplantations. However, numerous challenges persist in achieving the regeneration of fully functional kidney tissue using these methods. Consequently, there is an ongoing demand for the development of new therapeutic strategies to address CKD effectively. One promising avenue for tissue regeneration is cell-based therapy, which has been explored through preclinical and clinical trials [67, 68]. More recently, extracellular vesicles (EVs) derived from mesenchymal stem cells (MSC) have gained recognition for their therapeutic potential, exhibiting characteristics similar to those of their parent cells and offering an alternative to traditional cell-based therapeutics [69–71]. Notably, kidney tissues are known to cease differentiation and growth after embryonic development [72], making it crucial to introduce suitable kidney progenitor cells for successful kidney regeneration. While various types of stem cells and kidney progenitor cells have shown promise in regenerating kidney tissues, they have often encountered limitations, resulting in the partial restoration of kidney functions [73]. Our research has focused on the cells involved in kidney development. In mammalian development, the IM arises from the primitive steak and subsequently differentiates into urogenital structures [74, 75]. IM exhibits multipotent properties that enable it to differentiate into two specific kidney progenitor cells, MM and ureteric bud, which play pivotal roles in the nephron and collecting duct formation, respectively (Figure S8). To address the challenges associated with the isolated use of specific kidney progenitor cells, we propose the utilization of IM, which has the potential to differentiate into kidney progenitor cells. Furthermore, by optimizing the conditions, we can induce IM to differentiate into renal progenitor cells, mirroring the natural differentiation of IM into MM and UE during the development process.

It is widely recognized that scaffold orientation plays a pivotal role in promoting cell migration, thus contributing to enhanced tissue regeneration. In our study, we employed porous scaffolds based on poly(lactic-co-glycolic acid) (PLGA) to load bioactive components and cells, aiming for sustained signal induction and mechanical stability. PLGA, a biodegradable polymer, has gained prominence as a matrix material for regenerative medicine due to its exceptional mechanical properties, adjustable degradability, and versatile processability [49]. Nevertheless, challenges persisted, including inflammatory responses in surrounding tissues due to acidic byproducts and limited bioactivity, which required solutions. In a previous study, we developed PME scaffolds by incorporating ricinoleic acid-grafted magnesium hydroxide (Mg(OH)_2_-RA; MH-RA; M) and extracellular matrix (ECM; E) into PLGA (MW; 110 kDa, 50:50 of LA: GA; P) scaffolds to provide pH neutralization effects and biomimetic properties, respectively [24, 49]. With relatively high molecular weight of PLGA, pH was maintained due to critical neutralizing effects during long term degradation time. Additionally, we introduced alpha lipoic acid-modified zinc oxide (ZnO-ALA; Z) into the PME scaffold to promote angiogenesis by releasing nitric oxide (NO) into body fluids. [76] ZnO-ALA exhibited angiogenic properties with a long-term NO-generating ability, facilitated by the combination of two mechanisms: the thiol/disulfide exchange reaction between an NO donor and ALA and the redox reaction of ZnO with the NO donor [33].

The angiogenic properties were further enhanced by the incorporation of ZnO and stimulated even more effectively with the inclusion of IM and dEV. Although further research is needed on the cell affinity according to the physical and biological properties of PLGA, the elimination of volatile organic solvents used in synthesis of PLGA-based scaffolds enable cell engraftment [77]. In addition to these functional improvements, PMEZ scaffolds exhibited mechanical properties similar to those of kidney tissues, aiding in the integration with kidney tissue during implantation.

Numerous approaches have been explored to regulate the quantity and quality of EVs released from cells, as the properties of EVs are intricately linked to the status of their parent cells [50]. To isolate EVs for promoting kidney differentiation of IM, conditioned media were collected during the IM differentiation process, which involved the use of differentiation factors such as FGF9, BMP7, RA, and heparin. While previous attempts have introduced IM differentiation into kidney progenitor cells using serum-enriched APEL medium [8], it became imperative to validate a serum-depleted media-mediated differentiation protocol to mitigate potential side effects from serum impurities. We optimized the IM differentiation protocol in chemically defined media (CDM) supplemented with differentiation factors. CDM has been employed to maximize production yield and harness the regeneration-related bioactivities of mesenchymal stem cell (MSC)-derived EVs without the interference of serum-derived impurities [56]. The expression patterns of renal markers exhibited similar trends following IM differentiation in CDM compared to the same processes in APEL media. Additionally, the contents of two representative kidney differentiation factors, FGF9 and BMP7, experienced significant enhancement within EVs (dEV) produced during the IM differentiation process toward kidney progenitor cells in CDM. This augmentation arises from the release of these components by differentiated cells during IM differentiation into MM and UE lineages [7, 45, 63].

Among the various differentiation possibilities for IM into distinct lineages, our studies have demonstrated that dEVs can facilitate the differentiation of IM into kidney progenitor cells of the metanephros lineage, both in two- and three-dimensional conditions, without the requirement for additional differentiation factors. Given the constrained spatial limitations and delivery methods within the in vivo environment, the ability to stimulate cell differentiation-mediated regeneration simply by introducing multicomponent EVs holds substantial promise and potential advantages in numerous aspects.

The regenerative potential of PMEZ/dEV/IM scaffolds in promoting kidney differentiation was demonstrated in a 3/4 nephrectomy nude mouse model designed to simulate CKD. To minimize immune-mediated responses involving human cells, immunodeficient nude mice successfully underwent surgical nephrectomy. In addition, bioactivities related to regeneration and kidney function restoration were assessed in the second and eighth weeks after the implantation of various types of scaffolds based on PMEZ. With the introduction of PMEZ/dEV/IM scaffolds, multiple factors associated with regeneration, including angiogenesis and anti-inflammation, displayed positive regulation, while fibrotic tissue dramatically diminished. Kidney fibrosis, characterized by the excessive deposition of extracellular matrix, as evidenced by Masson’s Trichrome analysis, represents a hallmark feature of various progressive CKD stages and often co-occurs with kidney defects such as tubule atrophy, inflammation, glomerulosclerosis, and fibrogenesis [64, 65].

Kidney injury molecule-1 (KIM-1), serving as a representative marker for kidney tissue damage, exhibited increased expression in the CKD model, reflecting elevated kidney fibrosis and inflammation, ultimately leading to a decreased number of podocytes. However, the expression level of KIM-1 was dramatically reduced with the implementation of PMEZ/dEV/IM scaffolds, suggesting an increase in the number of glomeruli as a result of tissue regeneration. This successful tissue regeneration led to the restoration of renal function to normal levels in the 3/4 nephrectomy mouse model. While the incorporation of dEV and IM promoted metabolic kidney activities, the synergistic effects of these two factors on renal functional recovery have yet to be elucidated, as the remaining 1/4 of the kidney tissue could play a role in restoring kidney functions. Cell tracking analysis will be essential to unravel the mechanisms behind kidney regeneration using introduced bioactive materials, and kidney restoration should be achieved in a severely injured CKD model to clearly verify the synergistic effects of IM and dEV on kidney differentiation and regeneration.

## Conclusion

Our study has demonstrated that bioactive PLGA-based scaffolds, containing multipotent cells capable of differentiating into various kidney progenitor cells along with several supporting components, facilitate kidney tissue regeneration in a 3/4 nephrectomy mouse model that mimics CKD. We utilized porous PLGA (P) scaffolds and incorporated antacid MH-RA (M), biomimetic acellular ECM (E), and nitric oxide (NO)-generating ZnO-ALA (Z) to create PMEZ scaffolds, which exhibited mechanical properties sufficient to integrate with the surrounding tissues following implantations. Given the limited capacity for renal cell regeneration, the incorporation of human pluripotent stem cell-derived IM cells, possessing the ability to differentiate into renal progenitor cells, is believed to support kidney tissue regeneration through differentiation within the scaffold system enriched with differentiation factor-loaded extracellular vesicles (dEV). The addition of PMEZ/dEV/IM scaffolds resulted in elevated levels of kidney differentiation and regeneration-related factors. The synergistic bioactivities of the distinct components within PMEZ/dEV/IM scaffolds provided an optimal microenvironment for the morphogenetic formation of kidney tissues and facilitated functional recovery in the 3/4 nephrectomy mouse model. Based on these promising results, our scaffold system represents a highly motivated strategy for kidney tissue engineering and regeneration, offering a potential emerging therapeutic approach for CKD.

### Electronic supplementary material

Below is the link to the electronic supplementary material.

**Additional file 1: Table S1:** List of primer sequences used for RT-qPCR in vitro (for human genes) **Table S2:** List of primer sequences used for RT-qPCR in vivo (for mouse genes) **Figure S1: **(A) Nitric oxide release profile analyzed with NO sensor and (B) standard curve of NO current depending on concentration **Figure S2:** (A) Cell images of cellular morphological changes resulting from IM differentiation. (B) The gene expression levels of renal differentiation factors by different medium (APEL and CDM) by RT-qPCR (n = 3). **Figure S3: **The fluorescent images for (A) dEV and ?(B) IM incorporation into PMEZ scaffolds using confocal microscopy. Green: DiO-labeled dEVs, Blue: Hoechst-labeled IM (Scale bars equal to 40 µm) **Figure S4:** (A) The gene expression levels of LHX1 in IMctrl, IMdF, and IMdEV_2D by RT-qPCR (n = 3). (B) The gene expression levels of LHX1 in IMctrl, IMdF, and IMdEV_3D by RT-qPCR (n = 3). **Figure S5:** (A) The fluorescence-based immunohistochemistry of AQP-1 expression of scaffolds at 2 weeks after implantations (Scale bar: 100 µm). (B) The fluorescence-based immunohistochemistry of Nephrin expression of scaffold at 2 weeks after implantations (Scale bar: 100 µm). **Figure S6:** The gene expression levels of pro-inflammatory factors (TNF-α and IL-8) on the mouse model at (A) 2 and (B) 8 weeks after implantations (n = 3). **Figure S7:** The fluorescence-based immunohistochemistry of KIM-1 expression of scaffolds at 2 weeks after implantations (Scale bars equal to 100 µm) **Figure S8: **The development of renal differentiation

## Data Availability

The datasets used and/or analysed during the current study are available from the corresponding author on reasonable request.

## List of abbreviations

CKD: Chronic kidney disease
ESKD: End-stage kidney disease
MM: Metanephic mesenchyme
UE: Ureteric epithelium
IM: Intermediate mesoderm
PSC: Pluripotent stem cell
PLGA (P): poly(lactic-*co*-glycolic) acid
Mg(OH)_2_-RA (M): Ricinoleic acid-grafted magnesium hydroxide
ECM (E): Extracellular matrix
NO: Nitric oxide
ZnO-ALA (Z): Alpha lipoic acid-conjugated zinc oxide
GSH: Glutathione
SNAP: S-Nitroso-N-acetylpenicillamine
EVs: Extracellular vesicles
APEL: Animal product-free medium
CDM: Chemically defined media (CellCor^™^ CD MSC)
MSC: Mesenchymal stem cell
dF: Differentiation factor
cEVs: Control extracellular vesicles
dEVs: Differentiating extracellular vesicles
FGF9: Human fibroblast growth factor 9
BMP7: Bone morphogenetic protein 7
RA: Retinoic acid
HEP: Heparin
CM: Culture media
TSG101: Tumor susceptibility gene 101
Apo-A1: Apolipoprotein A1
PAX2: Paired box 2
WT1: Wilms` tumor 1
SIX2: SIX Homeobox 2
HOXD11: Homeobox D11
HOXB7: Homeobox B7
LHX1: LIM Homeobox 1
GAPDH: Glyceraldehyde-3-phosphate dehydrogenase
NF-kB: Nuclear factor kappa B
TNF-a: Tumor necrosis factor alpha
IL-6: Interleukin-6
IL-8: Interleukin-8
IL-4: Interleukin-4
IL-1Ra: Interleukin 1 receptor antagonist
VEGF: Vascular endothelial growth factor
HGF: Hepatocyte growth factor
HIF-1a: Hypoxia-inducible factor 1-alpha
RT-qPCR: Reverse transcription-quantitative polymerase chain reaction
H&E: Hematoxylin and Eosin
MT: Massons`s Trichrome
DAPI: 4’,6-diamidino-2-phenylindole
AQP-1: Aquaporin-1
KIM-1: Kidney injury molecule 1
LTL: Lotus tetragonolobus lectin
CRP: c-Reactive protein
BUN: Blood urea nitrogen
