# Supplementary material for: Kidney tissue regeneration using bioactive scaffolds incorporated with differentiating extracellular vesicles and intermediate mesoderm cells
Source: Biomater Res. 2023 Dec 5;27:126. doi: 10.1186/s40824-023-00471-x (PMC10696796; doi:10.1186/s40824-023-00471-x)
Supplement: Supplementary file 1 — Additional file 1: Table S1: List of primer sequences used for RT-qPCR in vitro (for human genes) Table S2: List of primer sequences used for RT-qPCR in vivo (for mouse genes) Figure S1: (A) Nitric oxide release profile analyzed with NO sensor and (B) standard curve of NO current depending on concentration Figure S2: (A) Cell images of cellular morphological changes resulting from IM differentiation. (B) The gene expression levels of renal differentiation factors by different medium (APEL and CDM) by RT-qPCR (n = 3). Figure S3: The fluorescent images for (A) dEV and ?(B) IM incorporation into PMEZ scaffolds using confocal microscopy. Green: DiO-labeled dEVs, Blue: Hoechst-labeled IM (Scale bars equal to 40 µm) Figure S4: (A) The gene expression levels of LHX1 in IMctrl, IMdF, and IMdEV_2D by RT-qPCR (n = 3). (B) The gene expression levels of LHX1 in IMctrl, IMdF, and IMdEV_3D by RT-qPCR (n = 3). Figure S5: (A) The fluorescence-based immunohistochemistry of AQP-1 expression of scaffolds at 2 weeks after implantations (Scale bar: 100 µm). (B) The fluorescence-based immunohistochemistry of Nephrin expression of scaffold at 2 weeks after implantations (Scale bar: 100 µm). Figure S6: The gene expression levels of pro-inflammatory factors (TNF-α and IL-8) on the mouse model at (A) 2 and (B) 8 weeks after implantations (n = 3). Figure S7: The fluorescence-based immunohistochemistry of KIM-1 expression of scaffolds at 2 weeks after implantations (Scale bars equal to 100 µm) Figure S8: The development of renal differentiation [file 40824_2023_471_MOESM1_ESM.docx]

**Kidney tissue regeneration using bioactive scaffold incorporated with differentiating extracellular vesicle and**

**intermediate mesoderm cells**

*Seung-Gyu Cha,^1†^ Won-Kyu Rhim,^1†^ Jun Yong Kim,^1,2,3†^ Eun Hye Lee,^4^ Seung Yeon Lee,^1^ Jeong Min Park,^1^ Jeoung Eun Lee,^5^ Hyeji Yoon,^7^Chun Gwon Park,^2,3^ Bum Soo Kim,^4,6^Tae Gyun Kwon^4,6^ Youngmi Lee,^7^ Dong Ryul Lee^1,5^ and Dong Keun Han^1,*^*

^1^Department of Biomedical Science, CHA University, 335 Pangyo-ro, Bundang-gu, Seongnam-si, Gyeonggi-do 13488, Republic of Korea

*^2^Department of Biomedical Engineering and ^3^Intelligent Precision of Healthcare Convergence, SKKU Institute for Convergence, Sungkyunkwan University (SKKU),2066 Seobu-ro, Jangan-gu, Suwon-si, Gyeonggi-do 16419, Republic of Korea*

*^4^Joint Institute for Regenerative Medicine, Kyungpook National University, Jung-gu, Daegu 41944, Republic of Korea*

*^5^CHA Advanced Research Institute, Bundang Medical Center, CHA University, Sungnam-si, Gyeonggi-do 13488, Republic of Korea*

*^6^Department of Urology, School of Medicine, Kyungpook National University, Jung-gu, Daegu 41944, Republic of Korea*

*^7^Department of Chemistry and Nanoscience, Ewha Womans University, Seodaemun-gu, Seoul, Republic of Korea*

Submitted to

***Biomaterials Research***

2023. 10. 6

^†^These authors equally contributed to this work.

***Corresponding author:** dkhan@cha.ac.kr (D. K. Han)

**Supporting information**

**Table S1. List of primer sequences used for RT-qPCR *in vitro* (for human genes)**

| Target gene | Forward sequence (5’-3’) | Reverse sequence (5’-3’) |
| --- | --- | --- |
| PAX2 | GCA ACC CCG CCT TAC TAA T | AAC TAG TGG CGG TCA TAG GC |
| WT1 | GCG GAG CCC AAT ACA GAA TA | GAT GCC GAC CGT ACA AGA GT |
| SIX2 | CGC CCA TGT GGG TCA GTG GG | AGC CGG GAG CGC TGT AGT CA |
| HOXD11 | GCC AGT GTG CTG TCG TTC CC | CTT CCT ACA GAC CCC GCC GT |
| HOXB7 | GCC TAC AAA TCA TCC GGC CA | GGT TGG AAG CAA ACG CAC AA |
| GDNF | CCA ACC CAG AGA ATT CCA GA | AGC CGC TGC AGT ACC TAA AA |
| CRET | CTC GAC GAC ATT TGC AAG AA | AGC ATT CCG TAG CTG TGC TT |
| OSR1 | GGA CCT CTG CGG AAC AAG | TGC AGG GAA GGG TGG ATA |
| LHX1 | ATG CAA CCT GAC CGA GAA GT | CAG GTC GCT AGG GGA GAT G |
| 18 s | GCA ATT ATT CCC CAT GAA CG | GGG ACT TAA TCA ACG CAA GC |

**Table S2. List of primer sequences used for RT-qPCR *in vivo* (for mouse genes)**

| Target gene | Forward sequence (5’-3’) | Reverse sequence (5’-3’) |
| --- | --- | --- |
| IL-1RA | TGC ACA ACA CTA GAG GCT GA | AGT GAT CAG GCA GTT GGT GA |
| IL-4 | TCA ACC CCC AGC TAG TTG TC | TGT TTC TTC GTT GCT GTG AGG |
| IL-6 | AGTTGCCTTCTTGGGACTGA | TCCACGATTTCCCAGAGAAC |
| IL-8 | GCC AAC AGT AGC CTT CAC CCA T | GGT GAT ATT CGA GACCAT TTA CTG |
| HGF | AAC GCG GAT GGT TTA TTA CG | TGG TGC TGA CTG CAT TTC TC |
| HIF-α | GGG GAG GAC GAT GAA CAT CAA | GGG TGG TTT CTT GTA CCC ACA |
| HOXB7 | ATC TAC CCC TGG ATG CGA AGC | CCG AGT CAG GTA GCG ATT CTA G |
| NF-κB | TGG TTC ATC GGG CTC ATG T | TTG ATG AAG CAG ACG ATG AG |
| Pax2 | AAA TCT CTA TGC AAA ATG ACG AAC AT | GAG AGA TGC AGG GCG ATG AA |
| SIX2 | CCG CGA GCT CTA CAAAAT CC | CTT CTC CGC CTC GAT GTA GT |
| TNF-α | TTCACTGGAGCCTCGAATGT | ACCTGACCACTCTCCCTTTG |
| VEGF | AGC ACA GCA GAT GTG AAT GC | TTT CTT GCG CTT TCG TTT TT |

**Figure legends of supporting information**

**Figure S1.** (A) Nitric oxide release profile analyzed with NO sensor and (B) standard curve of NO current depending on concentration

**Figure S2.** (A) Cell images of cellular morphological changes resulting from IM differentiation. (B) The gene expression levels of renal differentiation factors by different medium (APEL and CDM) by RT-qPCR (n = 3).

**Figure S3.** The fluorescent images for (A) dEV and (B) IM incorporation into PMEZ scaffolds using confocal microscopy. Green: DiO-labeled dEVs, Blue: Hoechst-labeled IM (Scale bars equal to 40 μm)

**Figure S4.** (A) The gene expression levels of LHX1 in IM_ctrl_, IM_dF_, and IM_dEV_2D_ by RT-qPCR (n = 3). (B) The gene expression levels of LHX1 in IM_ctrl_, IM_dF_, and IM_dEV_3D_ by RT-qPCR (n = 3).

**Figure S5.** (A) The fluorescence-based immunohistochemistry of AQP-1 expression of scaffolds at 2 weeks after implantations (Scale bar: 100 μm). (B) The fluorescence-based immunohistochemistry of Nephrin expression of scaffold at 2 weeks after implantations (Scale bar: 100 μm).

**Figure S6.** The gene expression levels of pro-inflammatory factors (TNF-α and IL-8) on the mouse model at (A) 2 and (B) 8 weeks after implantations (n = 3).

**Figure S7.** The fluorescence-based immunohistochemistry of KIM-1 expression of scaffolds at 2 weeks after implantations (Scale bar: 100 μm

**Figure S8.** The development of renal differentiation.

**Figure S1**

**
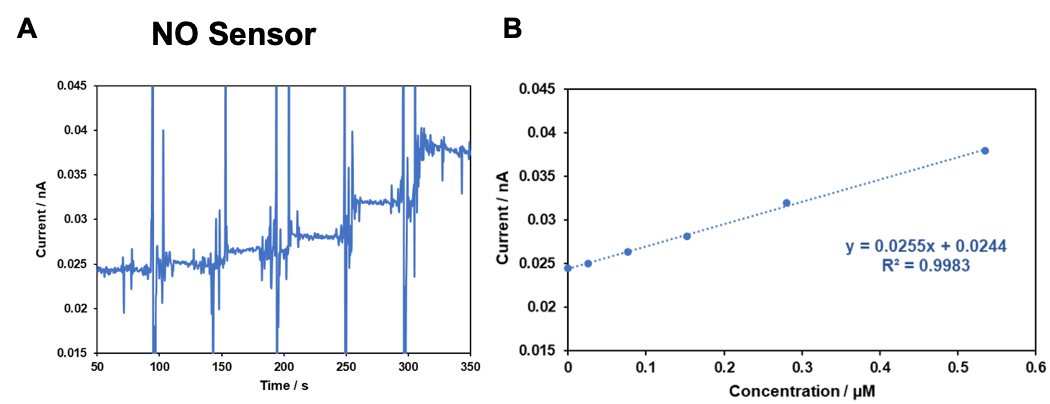
**

**Figure S2**


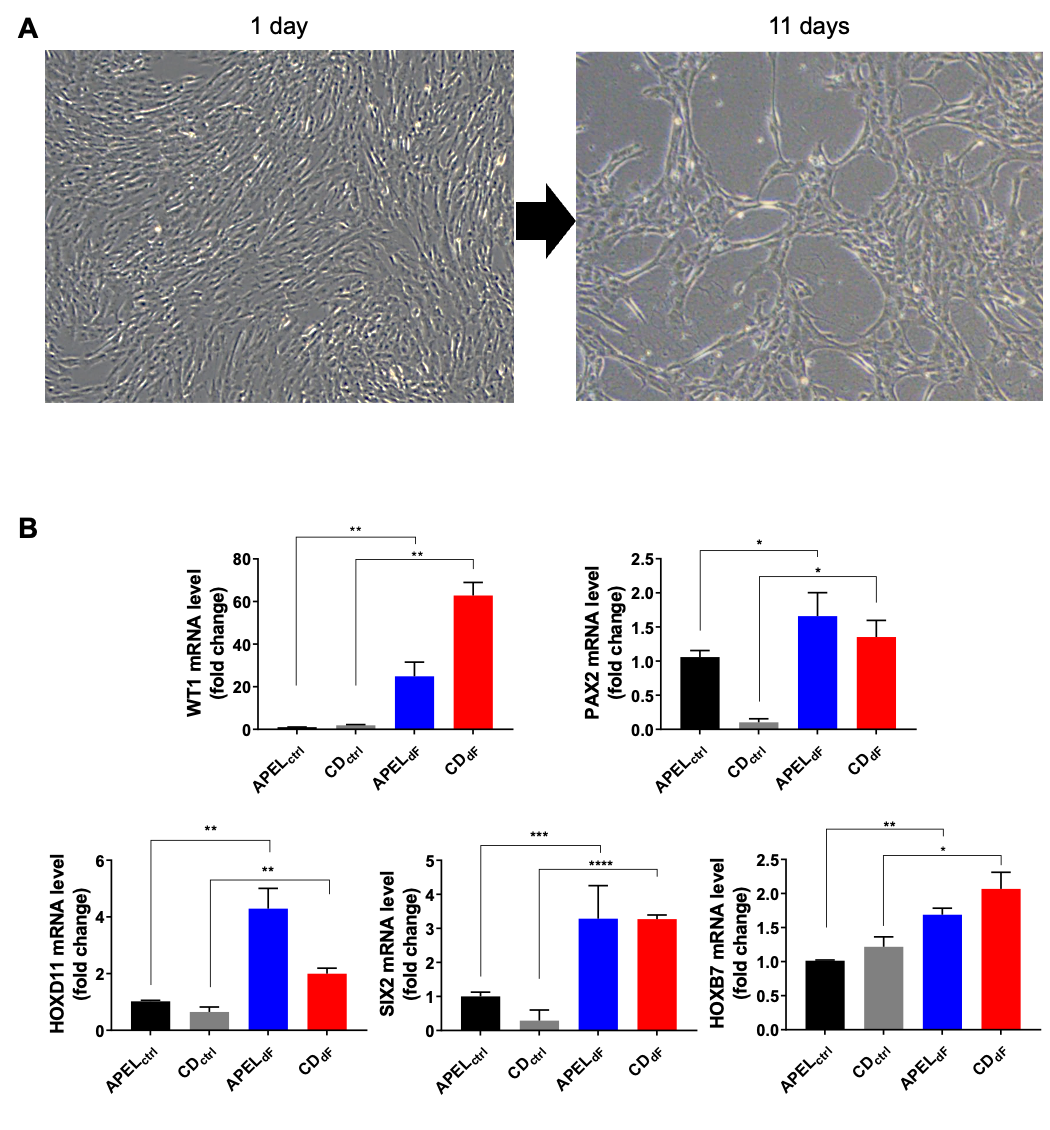


**Figure S3**

**
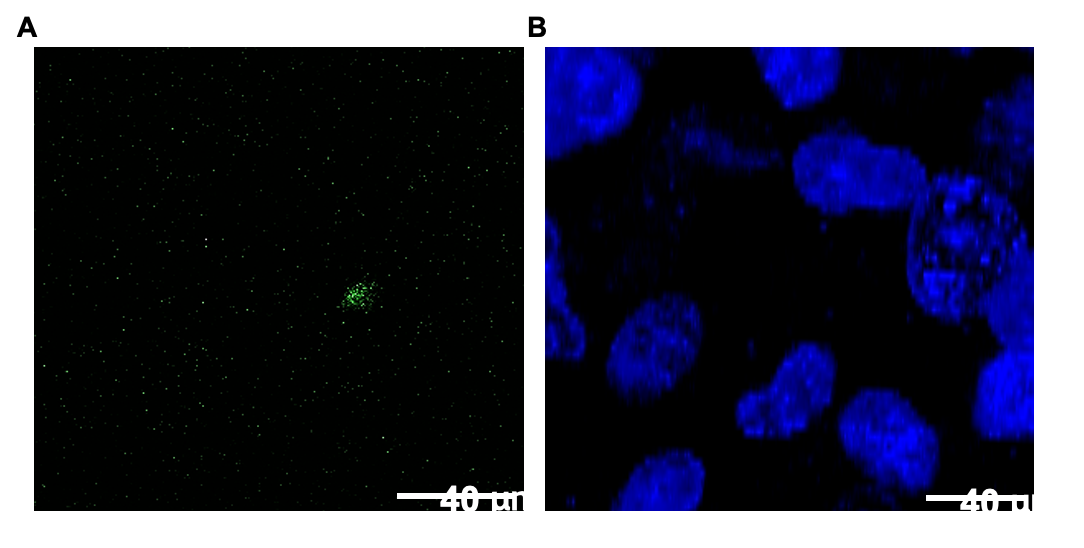
**

**Figure S4**

**
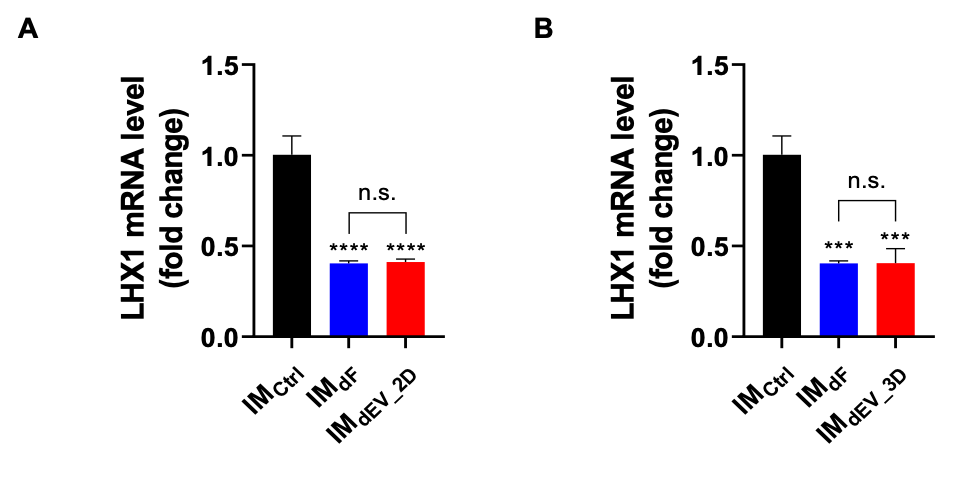
**

**Figure S5**


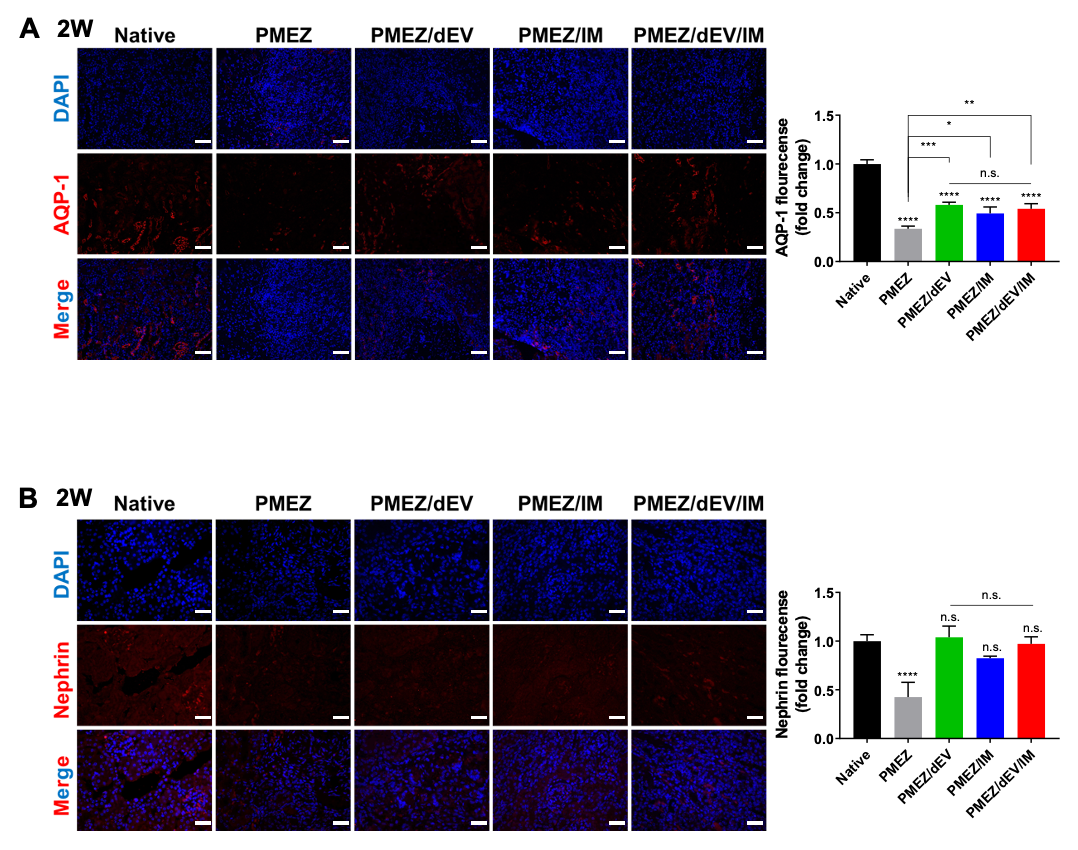


**Figure S6**

**
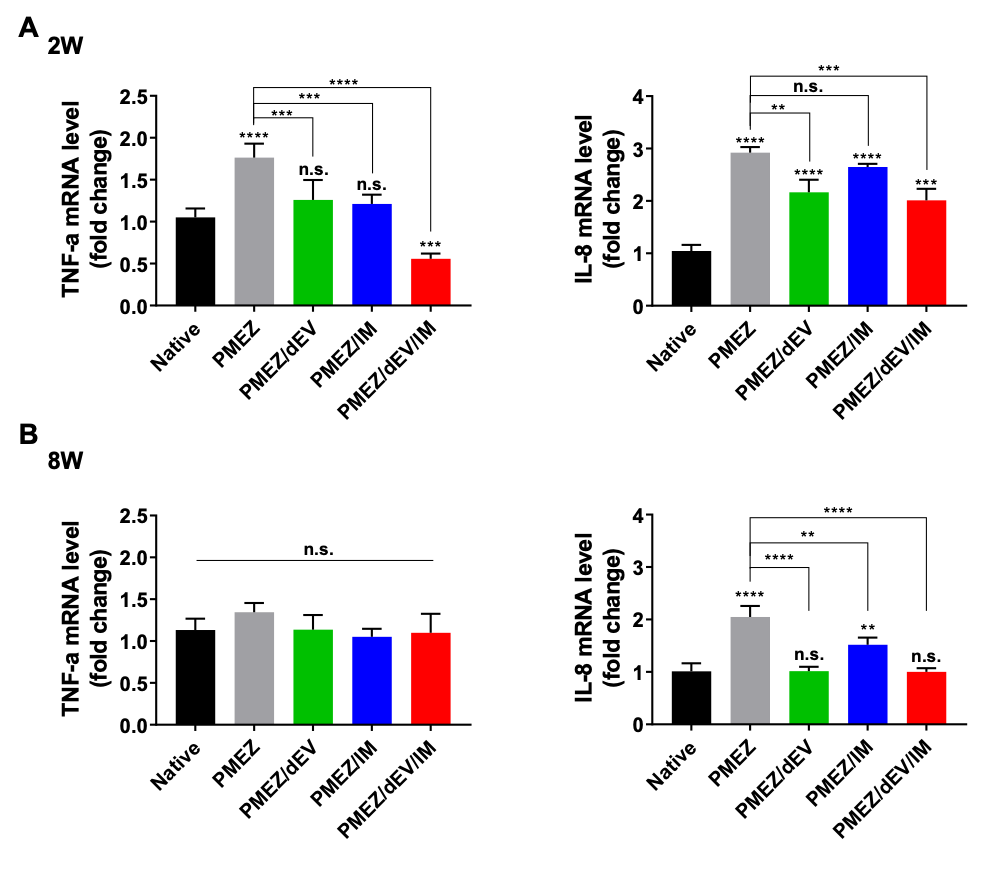
**

**Figure S7**

**
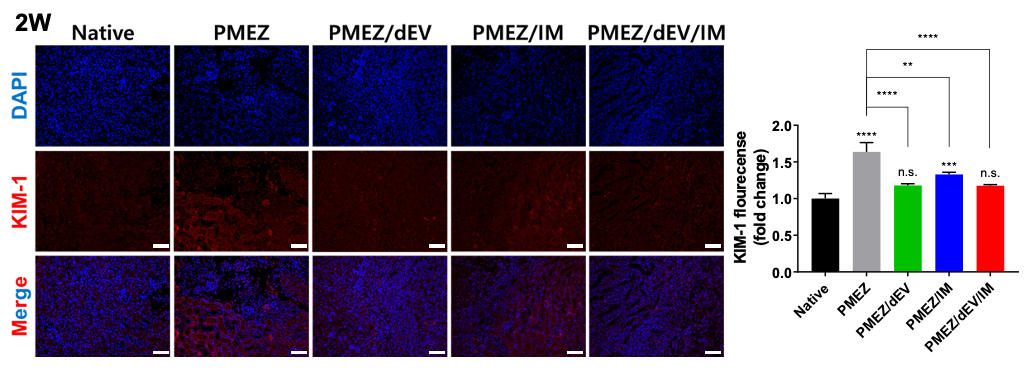
**

**Figure S8**

**
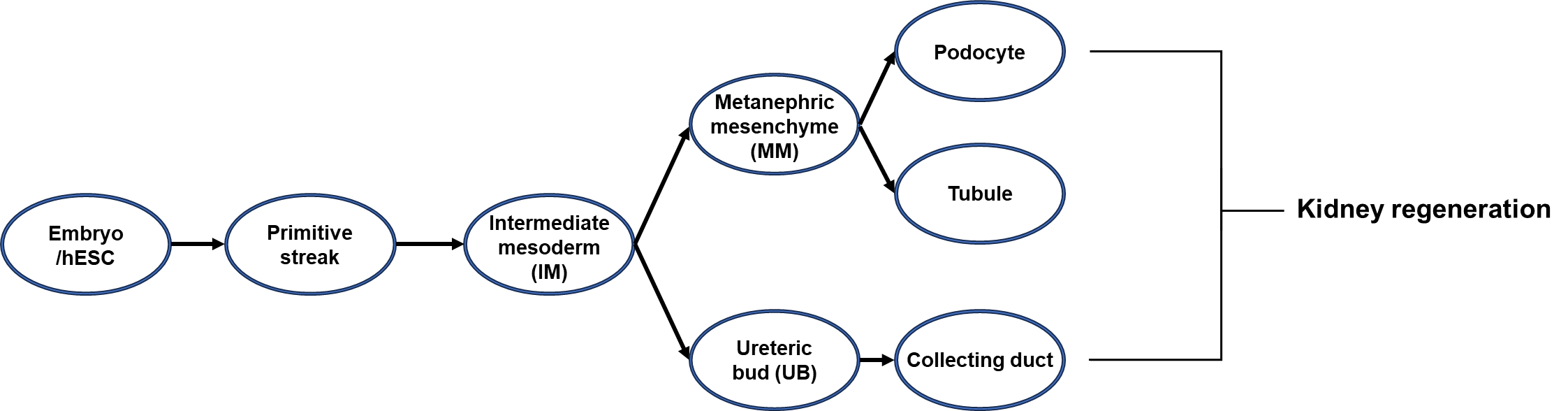
**
